# Positional cloning of a peanut CC-NBS-LRR gene, *AhRRS6*, confers resistance to *Ralstonia solanacearum*

**DOI:** 10.3389/fpls.2025.1718434

**Published:** 2026-02-03

**Authors:** Huiwen Fu, Yuhui Zhuang, Chong Zhang, Shipeng Li, Yongli Zhang, Wenzhi Lu, Lihui Wang, Sheidu Abdullaziz, Yuting Chen, Tiecheng Cai, Qiang Yang, Xiangyu Chen, Rajeev K. Varshney, Zujian Wu, Hua Chen, Weijian Zhuang

**Affiliations:** 1 College of Plant Protection, Center for Legume Plant Genetics and Systems Biology, Fujian Agriculture and Forestry University, Fuzhou, Fujian, China; 2 Center for Legume Plant Genetics and Systems Biology, College of Agronomy, Fujian Agriculture and Forestry University, Fuzhou, Fujian, China; 3 Center for Legume Plant Genetics and Systems Biology, College of Life Science, Fujian Agriculture and Forestry University, Fuzhou, Fujian, China; 4 Department of Agronomy, Faculty of Agriculture, Nasarawa State University, Keffi, Nasarawa State, Nigeria; 5 Centre for Crop and Food Innovation, State Agricultural Biotechnology Centre, Food Futures Institute, Murdoch University, Murdoch, WA, Australia

**Keywords:** *AhRRS6*, bacterial wilt resistance, BSR-seq, functional characterization, peanut, *Ralstonia salanacearum*

## Abstract

Bacterial wilt, caused by *Ralstonia solanacearum*, is a destructive disease with no effective chemical control, severely affecting global crop production. This study applied BSR-seq on 581 recombinant inbred lines (RILs), combined with linkage mapping, to identify resistance quantitative trait loci (QTL). Illumina sequencing yielded 189.6 Gb of data, identifying 70,035 high-quality SNPs from 55,840 genes. Two resistance loci were mapped on chromosome 12: a novel 1.11 Mb QTL and an adjacent 1.03 Mb region. Five CC-NBS-LRR-type resistance candidate genes were identified. The *AhRRS6* alleles were cloned, and three allele-specific SNP markers were developed and validated across peanut breeding varieties. Additionally, 3,851 differentially expressed genes were detected, including key resistance-related genes. Transgenic *AhRRS6y* conferred strong resistance to *R. solanacearum*, while *AhRRS6x* caused susceptibility in both *Nicotiana benthamiana* and *Arabidopsis thaliana*. These alleles differentially regulated genes in HR, ETI, and PTI pathways, particularly affecting *NbPDF1.2* and *NbNDR1*. *AhRRS6y* expression reduced oxidative damage, indicated by lower malondialdehyde and higher ascorbate peroxidase activity. This work provides critical genetic resources for breeding bacterial wilt-resistant peanut varieties and enhances the mechanistic understanding of plant immune responses.

## 1 Introduction

The cultivated peanut (*Arachis hypogaea* L.) is an important oilseed and food crop grown globally. However, one of the major threats to peanut production is bacterial wilt (BW), caused by *Ralstonia solanacearum (Rs)*, a soil-borne pathogen that infects a wide range of economically important plants, including eggplant, pepper, tobacco, and tomato (Zhang et al., 2017). BW can cause substantial yield and quality losses, ranging from 10% to 30% in peanuts. Given the limited effectiveness of biological and agronomic control methods, breeding resistant varieties has emerged as the most viable strategy to combat this disease (Wang et al., 2024). Achieving this goal requires the identification of resistance genes and understanding the underlying resistance mechanisms.

To date, many quantitative trait loci (QTLs) associated with resistance to *Rs* have been successfully identified in various crop species, such as pepper (Du et al., 2019), eggplant (Lebeau et al., 2013; Salgon et al., 2017), and tomato (Thoquet et al., 1996; Wang et al., 2013; Shin et al., 2020). In peanut, several QTLs linked to bacterial wilt resistance (BWR) have been discovered, with notable findings on linkage groups LG1 and LG10. A major QTL for resistance on LG1 in the wild B02 chromosome has been associated with two peaks, and related SNP markers were developed from crosses between resistant (Yueyou92) and susceptible (Xinhuixiaoli) varieties (Zhao et al., 2016). Another stable QTL, *qBWRB02.1*, was identified on chromosome B02 using195 recombinant inbred lines (RILs) from a cross between Yuanza 9102 and Xuzhou 68-4 (Luo et al., 2019), and further refined into two sub-QTLs, *qBWRB02-1-1* (2.81-4.24 Mb) and *qBWRB02-1-2* (6.54-8.75 Mb), corroborating Zhao’s findings (Luo et al., 2020). Additionally, a genomic region on chromosome 12 (1.8–9.0 Mb) has been significantly associated with Rs resistance in peanuts (Zhang et al., 2022). Despite these efforts, functional resistance genes in peanut remain largely uncharacterized. In contrast, map-based cloning in other crops has successfully identified resistance genes, such as *RRS1-R* and *ERECTA* in *Arabidopsis*. *RRS1-R*, a typical TIR-NB-LRR resistance gene, activates downstream defense genes upon recognition of the bacterial effectors PopP2, triggering an effector-induced resistance mechanism (Deslandes et al., 2002). Similarly, *ERECTA*, a receptor-like kinase gene, confers disease resistance through phosphorylation of downstream genes (Godiard et al., 2003). In peanut, two resistance genes, *AhRRS5* (an NBS-LRR gene) and *AhRLK1* (a receptor-like kinase), have been identified through transcriptomic analysis, and their heterologous expression in tobacco enhanced resistance to BW (Zhang et al., 2017, 2019). However, the positional cloning of key resistance genes to BW has not yet been reported in peanut.

Plants have evolved a sophisticated multilayered immune system to defend against pathogens (Jones and Dangl, 2006; Zhou and Zhang, 2020). The initial defense layer, termed pathogen-associated molecular pattern (PAMP)-triggered immunity (PTI), is activated when plant receptors recognize conserved microbial PAMPs. PTI responses include the production of reactive oxygen species (ROS), activation of the MAPK cascade, and the upregulation of pathogenesis-related genes (Dodds and Rathjen, 2010). However, pathogens can overcome PTI by secreting effector proteins (Büttner, 2016; Varden et al., 2017), some of which are recognized by intracellular NLRs (nucleotide-binding leucine-rich repeat receptors), triggering effector-triggered immunity (ETI). ETI is a more intense and prolonged immune response, often accompanied by localized programmed cell death (PCD) at the infection site, known as the hypersensitive response (HR). Both PTI and ETI contribute to local and systemic immunity, the latter being termed systemic acquired resistance (SAR), which protects uninfected tissues from subsequent pathogen attacks (Fu and Dong, 2013). As central components of ETI, NLRs rapidly activate defense responses upon recognizing pathogen effectors. Therefore, identifying NLR genes conferring resistance to BW is essential for developing strategies to enhance plant immunity. Recent advances in Bulk Segregant RNA Sequencing (BSR-Seq), which targets expressed genes linked to specific traits, have shown promise in identifying disease resistance markers (Xie et al., 2020). This technique has been successfully applied in several crops, including wheat (Wu et al., 2018), maize (Du et al., 2017), soybean (Huang et al., 2024), and sugarcane (Wu et al., 2022).

In this study, we employed BSR-Seq to analyze a population of 581 F_13_ progenies derived from a cross between the resistant parent YY92 and the susceptible parent XHXL. By integrating ΔSNP-index association analysis and Euclidean distance (ED) methods, and further validating the results through QTL-seq, we identified a key QTL and three SNP markers associated with bacterial wilt resistance. These findings were confirmed using a panel of 22 accessions with distinct resistance profiles. Functional validation confirmed that the resistant allele *AhRRS6y* enhances *Rs* resistance in transgenic plants while *AhRRS6x* does not. The allelic variants differentially regulated HR/ETI/SA/PTI pathway markers, including *NbPDF1.2* and *NbNDR1*. We further showed that *AhRRS6y*-transgenic *Nicotiana benthamiana (Nb)* exhibited reduced oxidative damage through suppressed malondialdehyde accumulation and sustained ascorbate peroxidase induction. Our findings provide novel genetic targets for breeding BW-resistant peanut varieties and lay the groundwork for elucidating resistance mechanisms in legume crops.

## 2 Materials and methods

### 2.1 Plant material

The peanut breeding line Yueyou 92 (YY92), developed by the Guangdong Academy of Agricultural Sciences, China, was used as the resistant parent against peanut bacterial wilt, while the Chinese landrace Xinhuixiaoli (XHXL) was chosen as the susceptible parent. A Recombinant Inbred Line (RIL) population consisting of 581 lines at the F13 generation was derived from the cross Yueyou 92 × Xinhuixiaoli, utilizing the single seed descent (SSD) method for trait mapping associated with BWR. All RILs and parental lines were cultivated in a field located in Yangzhong County, Sanming, Fujian, China. Seeds of *Nb* and *Arabidopsis thaliana* ecotype *Col-0* were provided by our laboratory.

### 2.2 Pathogen inoculation

A total of 581 RILs were evaluated for BWR in three independently replicated field trials spanning three consecutive growing seasons: spring and autumn 2016 (2016S, 2016A), and spring 2017 (2017S). RILs from the F_11_-F_13_ generations and parental lines were inoculated with *Rs* isolates at 30–40 days post-emergence. Peanut seedlings were inoculated on the third and fourth leaves from the apex. Using a sterile blade, a perpendicular cut reaching two-thirds toward the midrib was made on each leaflet, with four leaflets inoculated per plant. Two uncut leaflets from inoculated leaves were collected at designated time points for analysis. For *Nb*, T_3_ transgenic plants at the 4–6 leaf stage were used, with wild-type plants as controls. For *Arabidopsis thaliana*, T_3_transgenic plants grown in potting mix for 20–30 days served as experimental materials, with ecotypes *Col-0* (susceptible) and *Nd-1* (resistant) as controls. Root inoculation was performed by making 1-cm lateral cuts on both sides of tobacco plants using sterilized scissors. Cuts extended one-third of the diagonal length and half the soil depth in seedling trays to expose roots. A 5-mL *Rs* suspension was applied to wounds. The inoculation method followed established protocols (Zhao et al., 2016; Zhang et al., 2017).

Virulent *Rs* strains *Rs-P.362200* (peanut), *Rs-GMI1000* (tobacco), and *Rs-FJ1003* (Arabidopsis) were cultured on TTC agar medium (0.5 g/L 2,3,5-triphenyltetrazolium chloride; 5 g/L peptone; 0.1 g/L casein hydrolysate; 2 g/L D-glucose; 15 g/L agar) at 28 °C for 48 hr. Bacterial suspensions were prepared in sterile 0.02% Tween-20 water, adjusted to OD_600_ = 0.5 (NanoDrop 2000c; Thermo Fisher Scientific), corresponding to ∼10&^8^ CFU/mL.

### 2.3 Resistance phenotyping

RILs were phenotyped 25 days post-inoculation using a 0–5 disease severity scale (Zhang et al., 2022), where higher values indicate increased susceptibility. Transgenic plants were assessed with species-specific indices: 0–4 for Nb and 0–9 for A. thaliana (Zhang et al., 2017). The disease index (DI) was calculated using the following formula:


$$\text{Disease index}=\frac{\sum_{0}^{5}x_{i}y_{i}}{x_{\max}\sum y_{i}}\times 100\%$$

Where;

x_i_: disease grade value, x_max_: the highest disease grade value, y_i_: the number of diseased plants corresponding to the disease rating. The average DI was calculated for the three replications in a single environment. Statistical analysis of variance (ANOVA) was conducted using the DPS7.5 software (Date Processing System, Science Press, China), Values are presented as the mean ± standard deviation or standard error, as indicated. Differences between groups were assessed using one-way ANOVA and statistical significance was set at *p* < 0.05.

### 2.4 Library construction and RNA sequencing

The average DI for each RIL was calculated based on phenotyping data from the 2016 spring (2016S), 2016 autumn (2016A), and 2017 spring (2017S) seasons. 30 resistant and 30 susceptible lines were then chosen to construct the extreme resistant/susceptible (R/S) pool. Total RNA was extracted from 124 samples (including two parents, 30 highly resistant progenies, and 30 susceptible progenies, both before and after inoculation) using the CTAB method with some modifications (Sharif et al., 2021).

The RNAs were treated with RNase-free DNase I (Takara, Dalian, China) to eliminate any contaminating genomic DNA. cDNA libraries were prepared using Illumina Paired End Sample Prep Kit. Paired-end reads (151 bp) from four libraries were generated using the Illumina HiSeq 2500 platform (Beijing Baimaike Biotechnology Co., Ltd., China) with a sequencing depth of approximately 15× of the cultivated peanut genome (~2.7 Gb) for each pool and about 5× for parental plants. The raw sequencing data of the eight libraries have been deposited in the NCBI Sequence Read Archive (SRA) under the BioProject. The raw sequencing data were filtered using Trimmomatic software to remove adapter reads, low-quality reads, joint sequences, and ribosomal RNAs, resulting in high-quality reads. The quality of the clean reads was assessed using FastQC (Bolger et al., 2014). The STAR (v2.3.0e) software (Dobin et al., 2013) was utilized to align the clean reads with the cultivated tetraploid peanut (*Arachis hypogaea subsp. fastigiata* var. *vulgaris*) cultivar ‘Shitouqi’ reference genome (Zhuang et al., 2019), obtaining mapped reads for subsequent analysis.

### 2.5 SNP detection and identification of candidate genomic regions

SNP calling was performed using GATK v3.1–1 software (McKenna et al., 2010), followed by initial filtering of high-quality SNP variants prior to association analysis. Filtering criteria included excluding polymorphic SNP sites with multiple genotypes and SNP sites exhibiting consistent genotypes between mixed pools and SNP sites, where recessive mixed pool genes did not originate from recessive parents. The SNP index between resistant and susceptible parents and bulks for each SNP was calculated (Takagi et al., 2013) using the following formula:


$$\text{SNP}-\text{index}(aa)=\frac{Maa}{Maa+Paa}$$

$$\text{SNP}-\text{index}(ab)=\frac{Mab}{Mab+Pab}$$

Δ SNP−index=SNP−index(aa)−SNP−index(ab)

ΔSNP-index was calculated by subtracting the SNP-index of the R-bulk from the SNP-index of the S-bulk. SNP-index plots were generated using sliding window analysis with a window size of 2 Mb and increments of 50 Kb. The closer the ΔSNP-index is to 1, the stronger the association of the SNP marker with the trait. The threshold for SNP screening was set as a test of 100,000 permutations coupled with 90.0% confidence level. Candidate regions exhibiting a ΔSNP-index value exceeding the threshold were identified as potential loci associated with bacterial wilt in peanuts. To further verify the result of the ΔSNP-index, the Euclidean distance (ED) algorithm was also used to calculate the candidate region based on the method proposed by (Trapnell et al., 2010).To eliminate background noise, the original ED value was processed to (ED)2 (Altschul et al., 1997), which was considered as the correlation value. The median plus 3 timers the standard deviation of the fitted values for all loci was taken as the correlation threshold for analysis (Trapnell et al., 2010). ED values were calculated using the formula below:


$$\text{ED}=\sqrt{{\left(A_{R-bulk}-A_{S-bulk}\right)}^{2}+{\left({\text{C}}_{R-bulk}-{\text{C}}_{S-bulk}\right)}^{2}+{\left({\text{G}}_{R-bulk}-{\text{G}}_{S-bulk}\right)}^{2}+{\left({\text{T}}_{R-bulk}-{\text{T}}_{S-bulk}\right)}^{2}}$$

The letters A, G, C and T represent the frequencies of corresponding cDNA nucleotides in resistant and susceptible populations. The higher the ED value, the stronger the correlation between variables and target characteristics. SNPeff (Cingolani et al., 2012) software was used to annotated the position and functional information of SNPs.

### 2.6 QTL-mapping of bacterial wilt resistance

To confirm the candidate regions for BRW, we performed traditional QTL analysis using existing genetic maps (Zhuang et al., 2019) on the 581 RIL population, focusing on the genomic regions identified by the QTL mapping method. The QTLs for bacterial wilt traits (2017 spring) were identified through composite interval mapping (CIM) analysis, incorporating three algorithms: HK (Haley-Knott regression), EM (Expectation-Maximization algorithm), and IMP (Interval Mapping), utilizing the R (4.2.0) qtl package and a permutation test (1,000 permutations, P = 0.05). A QTL was considered major and stable if its LOD value exceeded the cutoff and it had significant effects, accounting for more than 10% of the phenotypic variation.

### 2.7 Whole genome resequencing

From the peanut varieties used to construct the bulked samples, 10 resistant and 10 susceptible peanut varieties were randomly selected. Young leaves, ranging in size from 30 to 40 days, were collected for DNA extraction. The DNA extracts were separately used to construct Illumina sequencing libraries according to the manufacturer’s instructions. Paired-end sequencing of the libraries was performed using an Illumina HiSeq X Ten platform (Illumina). Raw sequencing reads were filtered to produce clean reads which were aligned to cultivated tetraploid peanut reference genome (Zhuang et al., 2019) using BWA (Li and Durbin, 2009). The alignment results were converted to bam format using SAM tools (Li et al., 2009), and duplicated reads were removed using the Picard package GATK (McKenna et al., 2010). Local realignment was performed to refine the read mapping in the presence of the variants, thereby generating a gVCF file. SNPs were filtered out using the following parameters: QD<2.0 || FS>60.0 || MQ<40.0 || SOR>3.0 || MQRankSum<-12.5 || ReadPosRankSum<8.0.

### 2.8 Functional annotation and enrichment analysis of the differentially expressed genes

Fragments per kilo bases of exon per million fragments mapped (FPKM) was utilized to calculate the expression level of functional genes mapped to the reference genome of cultivated peanut reference genome *A. hypogaea* var. Shitouqi (Zhuang et al., 2019) by using the Stringtie (Pertea et al., 2015) software. DEGs were defined as those with a fold change ≥ 2 and false discovery rate (FDR)< 0.01 using the EBseq v1.6.0 (Leng et al., 2013) software. Functional annotation of DEGs was conducted using information from http://peanutgr.fafu.edu.cn/Download.php. GO and KEGG pathway Enrichment analysis of the DEGs were implemented using the R package based on the previous studies (Yu et al., 2012). For GO analysis, GO term finder was used to describe the biological functions of a gene expression product (Boyle et al., 2004). For KEGG pathway analysis, the KEGG database was utilized to blast against the metabolic pathway.

### 2.9 Quantitative real-time PCR validation for differentially expressed genes

Real-time PCR for the relative expression level of DEGs was performed using ChamQ SYBR qPCR Master Mix (High ROX Premixed) (Vazyme, Nanjing, China) with specific primers (**Supplementary Table 20**) and *Ahactin* was used as an internal reference gene. All reactions were conducted in triplicate on an ABI7500 system. The relative expression levels of the DEGs were calculated using the comparative Ct method (2^-ΔΔCt^ method) (Schmittgen and Livak, 2008). The normalization was done by comparing the PCR threshold cycle number (Ct value) of the DEGs to that of the reference gene (*Ahactin*). The Student’s t-test was employed to compare differences between the control and experimental values.

### 2.10 Full-length cDNA cloning and vector construction

Total RNA was extracted from the leaves of resistant peanut YY92 and susceptible peanut XHXL to *Rs* using the CTAB method. Full-length *AhRRS6* cDNA was amplified with high-fidelity PCR using primers *AhRRS6-OE-F* and *AhRRS6-OE-R* (containing XbaI and SacI sites) (**Supplementary Table 20**), and cloned into the modified pBI121 vector, replacing the GUS gene. *AhRRS6* cDNA was also amplified with primers *AhRRS6-YFP-F* and *AhRRS6-YFP-R* (containing a BamHI site) (**Supplementary Table 20**) and cloned into the pFGC-eYFP vector, between the CaMV 35S promoter and EYFP gene, to create CaMV35S:: AhRRS6-YFP. CaMV35S::AhRRS6-YFP vectors were transformed into *Agrobacterium tumefaciens GV3101*, which was cultured in induction medium (10 mM methanesulfonic acid, pH 5.7, 10 mM MgCl_2_, 200 mM acetosyringone), diluted to OD_600_ = 0.8, and used to infiltrate *Nb* leaves with a needleless syringe. After 48 hours, YFP fluorescence was imaged using a laser confocal fluorescence microscope (Leica TCS SP8, Solms, Germany).

### 2.11 *Nicotiana benthamiana* and *Arabidopsis* transformation

*Nb* was used as the host for transformation with the CaMV35S::AhRRS6 fusion gene via the GV3101-mediated leaf-disc method (Rizhsky et al., 2002). For *Arabidopsis thaliana*, transgenic plants were generated using the floral dip protocol. T_0_, T_1_, and T_2_ progeny were selected on kanamycin-containing medium, with transgene integration confirmed by RT-PCR. Homozygous T_3_ lines were established for subsequent experiments.

### 2.12 Determination of MDA content and APX activity

At each time point, using the OE-*AhRRS6y* transgenic line as an example, three independent overexpression lines (OE-*AhRRS6y#1*, OE-*AhRRS6y#2*, and OE-*AhRRS6y#3*) were used. Each line was cultivated in three pots (with 18 plants per pot, each grown in an individual container) as three biological replicates, designated as OE-*AhRRS6y#1.1*, OE-*AhRRS6y#1.2*, OE-*AhRRS6y#1.3*, OE-*AhRRS6y#2.1*, OE-*AhRRS6y#2.2*, OE-*AhRRS6y#2.3*, OE-*AhRRS6y#3.1*, OE-*AhRRS6y#3.2*, and OE-*AhRRS6y#3.3*. For sampling, the second to third leaves were randomly collected from three plants in each of the following pot combinations and pooled into one bag: OE-*AhRRS6y#1.1*, OE-*AhRRS6y#2.1*, and OE-*AhRRS6y#3.1* for the first bag; OE-*AhRRS6y#1.2*, OE-*AhRRS6y#2.2*, and OE-*AhRRS6y#3.2* for the second bag; and OE-*AhRRS6y#1.3*, OE-*AhRRS6y#2.3*, and OE-*AhRRS6y#3.3* for the third bag. This process yielded three pooled samples, which were considered as three biological replicates for the OE-*AhRRS6y* transgenic line and used for APX and MDA assays. The same procedure was applied to the *OE-AhRRS6x* transgenic line. For the *Nb* control, three pots (with 18 plants per pot, each grown in an individual container) were used as three biological replicates, designated as *Nb-1*, *Nb-2*, and *Nb-3*. From each replicate, the second to third leaves of three randomly selected plants were collected and pooled into one bag, resulting in three pooled samples for APX and MDA content measurement. Error and significance were calculated using GraphPadPrism 8.0.1 software. For the data at each time point, we used one-way ANOVA to assess the overall differences among the three groups: *Nb*, OE-*AhRRS6y*, and OE-*AhRRS6x*. Upon confirming a significant difference, we further employed Student’s t-test to evaluate specific differences between each transgenic line and the control group (*Nb*).

Frozen samples were pulverized in liquid nitrogen. For MDA: 0.1 g tissue was homogenized in 0.5 mL PBS, which was centrifuged at 8,000 ×g rpm, for 10 min at 4 °C. Supernatant of 0.06 mL was mixed with 0.18 mL 0.5% TBA, which was then incubated at 95 °C, for 30 min. Later cooled on ice, and re-centrifuged at 10,000 ×g rpm, for 10 min, at 25 °C. Absorbance of 200 μL supernatant was measured at 532/600 nm in 96-well plates. MDA (nmol/g FW) = [ΔA_532-600_ × V_r_ × 10^9^/(155 × d)]/(M × V_s_/V_sa_) × F.

For APX: 0.1 g tissue was homogenized in 0.25 mL extraction buffer, centrifuged at 10,000 ×g rpm, for 10 min, at 4 °C. In 96-well plates, 20 μL supernatant was mixed with 140 μL PBS, 20 μL ascorbate, and 20 μL H_2_O_2_. ΔA_290_ was calculated from readings at 10s and 130s. APX activity (nmol/min/g FW) = [ΔA_290_ × V_r_ × 10^9^/(2.8 × d)]/(M × V_s_/V_sa_ × T) × F. V_r_: Reaction volume (0.24 mL MDA; 0.20 mL APX), d: Optical path (0.6 cm),V_s_: Sample volume used, V_sa_: Total supernatant volume, M: Sample mass (g), T: Reaction time (2 min for APX), F: Dilution factor.

## 3 Results

### 3.1 Phenotypic variation and transcriptome sequencing of extreme BWR bulks

**Figure 1:**
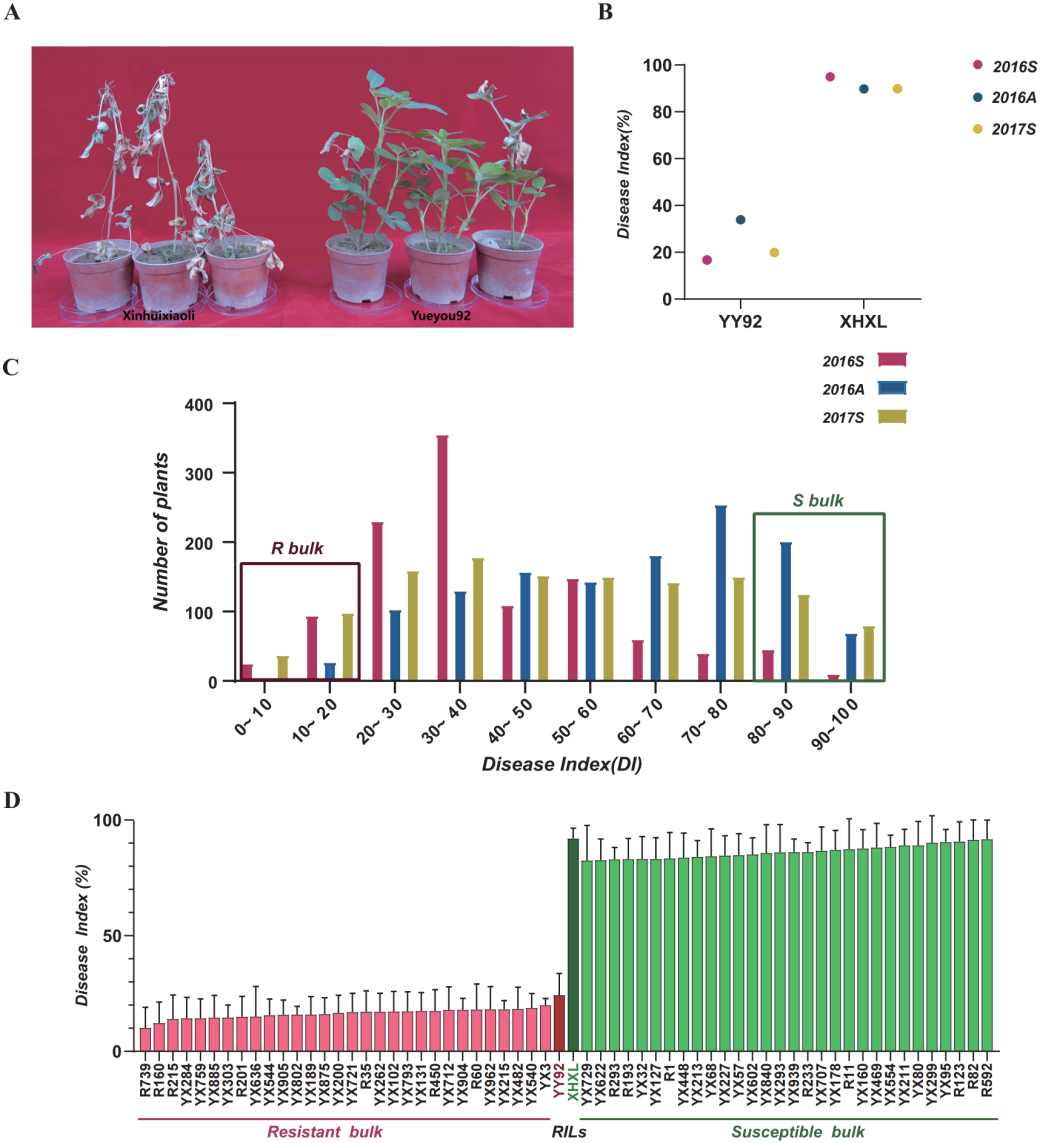
Phenotypic variations, disease index statistics, and construction of extreme bulks for resistance or susceptibility to *Rs* infection. **(A)** Phenotypic observation of resistant parent YY92 and susceptible parent XHXL after inoculation with *Rs*. **(B)** Disease index statistics for resistant parent YY92 and susceptible parent XHXL across three different crop seasons (2016_Spring, 2016_Autumn, and 2017_Spring). **(C)** Frequency distribution of disease indexes for RIL population at three different times. The y-axis represented the number of plants, whereas the x-axis represented the disease index. The red dashed box represented the resistant bulk (R-bulk), and the green dashed box represented the susceptible bulk (S-bulk). **(D)** Disease index of RIL population used to construct extreme bulks. Utilizing mean values obtained from three environments, each replicated thrice, the top 30 RILs with the lowest disease index and the bottom 30 RILs with the highest disease index were selected to form susceptible and resistant bulks, respectively.

To investigate the genetic basis of peanut resistance to *Rs*, we used the resistant variety YY92 (RP) and the susceptible variety XHXL (SP) as parental lines (**Figure 1A**). RILs population was developed and evaluated for resistance across three cropping seasons using a disease index (DI) to quantify symptom severity. DI value for YY92 was consistent and significantly lower than that of XHXL (**Figure 1B**). The DI distribution across 581 RILs showed continuous distribution with two peaks, indicating quantitative inheritance and the presence of major QTLs associated with BWR (**Figure 1C**). Based on phenotypic extremes, we selected two pools for bulked segregant analysis: the resistant bulk (R-bulk), consisting of 30 RILs with the lowest DI (10.22-20.00%) and the susceptible bulk (S-bulk), consisting of 30 RILs with the highest DI (81.68-92.79%) (**Figure 1D**, **Supplementary Table 1**). Each bulk and parental line was subjected to *Rs* inoculation and mock control to create treatment and control groups. We performed transcriptome sequencing on the parental and bulk DNA samples using Illumina HiSeq platform. After filtering low-quality reads, adapter sequences, and rRNA, we obtained clean data with a total of 14.58, 16.47, 10.24, 13.42, 32.37, 33.71, 36.35, and 32.49 gigabytes (Gb) of clean data across the eight samples. These clean datasets comprised 48,757,912; 55,072,811; 34,262,521; 44,888,427; 108,331,630; 112,881,894; 121,775,325 and 108,858,357 clean reads, respectively. The clean reads from all eight samples accounted for more than 97.72% of the total raw data, with a Q30 score exceeding 85.02%. The GC content of the clean reads ranged from 45.51% to 46.08%. We aligned the clean reads with the reference STQ peanut genome (Zhuang et al., 2019), and the mapping rates ranged from 78.17% to 81.07% (**Supplementary Table 2**). The high sequencing quality of the eight samples indicated that the data were sufficient for downstream analysis.

### 3.2 SNP calling and candidate gene identification for BWR

**Figure 2:**
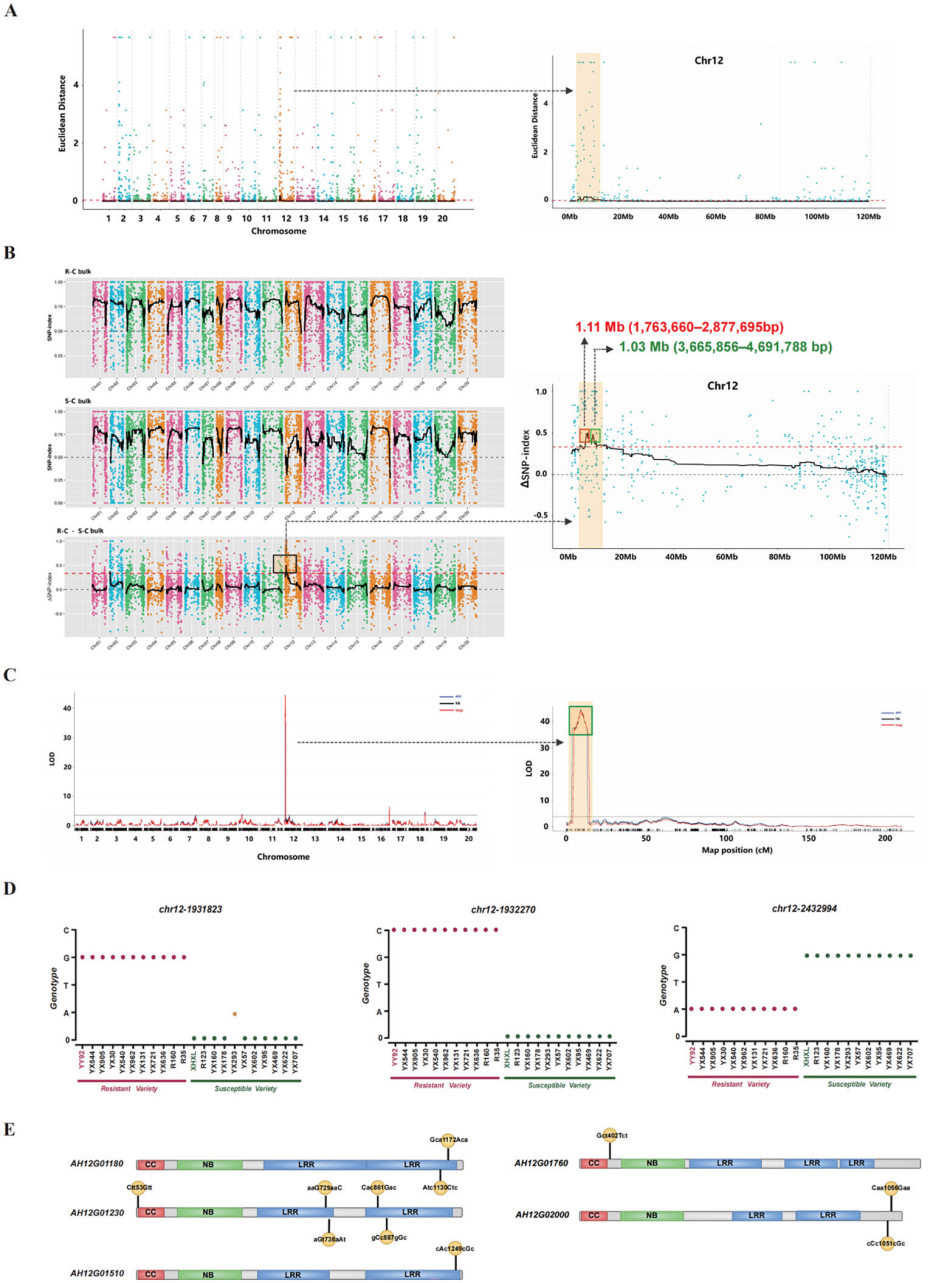
Identifying regions and genes associated with resistance to bacterial wilt disease and validating SNP markers through whole-genome resequencing. **(A)** Locating Candidate Regions Associated with Bacterial wilt Disease in Peanuts Using the ED method. The X-axis represents the names of chromosomes, with points of different colors representing ED values. The black line indicates the mean ED values within each window (2Mb windows sliding in 10 kb steps), while the red line represents the threshold line. **(B)** Locating Candidate Regions Associated with Bacterial wilt Disease in Peanuts Using the Delta SNP-Index Method. The X-axis represents the chromosome name. Each point of different colors represents an SNP locus. The black dashed line connects the mean SNP-index values within each window (2Mb windows sliding in 10 kb steps), and the red line represents the threshold line fitted through 1000 iterations to achieve a 90% confidence level. **(C)** QTL mapping was conducted based on an existing genetic map and phenotypic data from 2017 Spring, through composite interval mapping (CIM) analysis, incorporating three algorithms: HK (Haley-Knott regression), EM (Expectation-Maximization algorithm), and IMP (Interval Mapping).The LOD threshold was set at 3, and the results from the three algorithms were consistent. **(D)** The validated diagnostic markers for bacterial wilt resistance by whole genome re-sequencing. The X-axis represents 10 resistant varieties and 10 susceptible varieties selected based on disease index and subjected to whole genome re-sequencing. The Y-axis represents four nucleotide types: A, T, G, and C. The red dots represent the genotypes detected at the SNP locus in resistant varieties, while the green dots represent the genotypes at the SNP locus in susceptible varieties. The yellow dots represent no mutation detected at this locus. **(E)** The identified candidate NBS-LRR resistance proteins associated with bacterial wilt resistance. CC: Coiled-coil domain. NB: Nucleotide-binding site domain. LRR: Leucine-rich repeat domain. The positions of amino acid changes caused by nonsynonymous SNPs were shown in yellow.

To accurately identify genetic variants associated with BWR, SNP sites were called from RNA-seq data using the GATK software package. After filtering, 33,877 high-quality SNPs were retained from the treatment group and 36,158 from the control group (**Supplementary Tables 3**, **4**). For association analysis, we applied the ED algorithm to the control group. This analysis revealed three genomic regions with ED values greater than 0.02 (P< 0.01) (**Figure 2A**, **Supplementary Table 5**). Additionally, the ΔSNP-index analysis identified a major peak on chromosome 12 for BWR, with three distinct intervals showing a ΔSNP-index greater than 90% (**Figure 2B**; **Supplementary Figure 1**; **Supplementary Table 6**). Combining these results from both analyses, three high-confidence candidate regions were identified on chromosome 12: 1.15 Mb (1,763,660–2,912,931), 1.03 Mb (3,665,856–4,691,788), and 8.56 Mb (5,777,741–14,333,981) (**Supplementary Figure 2A**, **Supplementary Table 7**). Further *Rs* inoculation and ED analysis of the treatment group revealed two additional candidate intervals (**Supplementary Figure 3A**, **Supplementary Table 8**). Additionally, the SNP-index and ΔSNP-index methods identified two regions that deviated from the threshold, with a ΔSNP-index value at a 90% confidence level (**Supplementary Figure 3B**, **Supplementary Table 9**). By combining both approaches, two intersecting candidate regions were pinpointed for further analysis: 2.57 Mb (305,893–2,877,695) and 1.52 Mb (3,347,384–5,146,644) on chromosome 12 (**Supplementary Figure 2B**, **Supplementary Table 10**). Collectively, two QTL loci were identified at the 1.11 Mb (1,763,660–2,877,695 bp) and 1.03 Mb (3,665,856–4,691,788 bp) regions on chromosome 12. A total of 187 genes were located within these intervals, including 60 NBS-LRR genes (**Supplementary Table 11**). Meanwhile, we performed QTL mapping using a genetic map with 14,619 loci across 20 linkage groups (covering 3264.4 cM) (Zhuang et al., 2019) and BWR phenotypes from 581 RILs. The results revealed a significant QTL on chr12 (2847722bp - 6381141bp) with an LOD of 44.62, explaining 42.81% of the phenotypic variation (**Figure 2C**). This region overlaps with the (3,665,856–4,691,788 bp) region identified in BSR and corroborated in previous reports (Zhao et al., 2016 and Zhang et al., 2023). In contrast, the 1,763,660–2,877,695 bp region was uniquely identified by BSR-seq, representing a novel QTL discovery.

To further refine these candidate regions, we conducted a functional SNP analysis within the overlapping intervals. In the control group, we identified 103 SNPs within the target region. These included 10 intergenic SNPs, 27 intronic SNPs, 7 upstream SNPs, 2 downstream SNPs, 3 SNPs in the 5’ UTR, and 3 SNPs in the 3’ UTR. Among these, we also identified 13 synonymous and 34 nonsynonymous SNPs (**Supplementary Table 12**). In the treatment group, a total of 107 loci were identified within the same region, consisting of 9 intergenic, 22 intronic, 5 upstream, and 10 downstream SNPs. Furthermore, 2 SNPs were located in both the 5’ UTR and 3’ UTR regions, with 12 synonymous and 42 nonsynonymous SNPs (**Supplementary Table 13**). In total, 76 nonsynonymous mutations were identified in both groups located within 36 gene coding regions, including 16 NBS-LRR disease resistance genes. Among these, 11 loci were common to both datasets and affected five NBS-LRR resistance genes. These five genes includes, *AH12G01180, AH12G01230, AH12G01510, AH12G01760*, and *AH12G02000*, which were selected as candidate resistance genes associated with BW (**Figure 2E**, **Supplementary Table 14**).

### 3.3 Validation of SNP markers based on whole-genome resequencing

To assess the effectiveness of allelic SNP markers in distinguishing peanut resistance or susceptibility to *Rs*, we conducted validation analysis using 11 SNP loci located within the identified mapping intervals that caused non-synonymous mutations. For this purpose, whole-genome resequencing was performed on a panel of 20 peanut accessions, comprising 10 resistant and 10 susceptible genotypes (**Supplementary Table 15**). SNP detection revealed that 3 of 11 loci -Chr12-1931823, Chr12-1932270, and Chr12-2432994 - were reliably identified in the resequencing data (**Supplementary Table 16**). In all resistant varieties (YX544, YX905, YX30, YX540, YX962, YX131, YX721, YX636, R160, and R35), these loci displayed genotypes identical to the resistant parent (YY92). This pattern was also consistent with the genotypes observed in the resistant bulk (R-bulk) (**Figure 2D**). In contrast, among the 10 susceptible accessions (R123, YX160, YX178, YX293, YX57, YX602, YX95, YX469, YX622, and YX707), the Chr12–2432994 locus shared the same genotype as the susceptible parent, XHXL. This SNP was located within the *AH12G01510* gene. However, the other two loci (Chr12–1931823 and Chr12-1932270) were absent in both the susceptible parent and progeny (**Figure 2D**). Notably, these two loci were located within the coding region of the *AH12G01230* gene. Collectively, the consistent allelic patterns of these three SNPs across phenotypically divergent lines provide strong evidence of their utility as molecular markers for *Rs* resistance in peanut breeding programs.

### 3.4 Screening and functional enrichment analysis of differentially expressed genes in peanut

To investigate the global transcriptomic response to *Rs* infection, we performed differential gene expression (DEG) analysis. Substantial DEGs (fold change > 1.5, p< 0.01) were identified under *Rs* stress, in resistant and susceptible genotypes. The resistant parent (YY92) exhibited 9,303 DEGs (5,106 upregulated; 4,197 downregulated), while the resistant bulk showed 10,063 DEGs (5,644 upregulated; 4,419 downregulated). Comparative analysis revealed 1,140 consistently upregulated and 725 consistently downregulated genes shared between the resistant parent and bulk (**Supplementary Table 17**). In susceptible materials, the parent contained 9,705 DEGs (3,946 upregulated; 5,759 downregulated) while the bulk had 10,285 DEGs (4,872 upregulated; 5,413 downregulated), with 2,711 DEGs showing consistent differential expression (1,061 upregulated; 1,650 downregulated; **Supplementary Table 18**).

**Figure 3:**
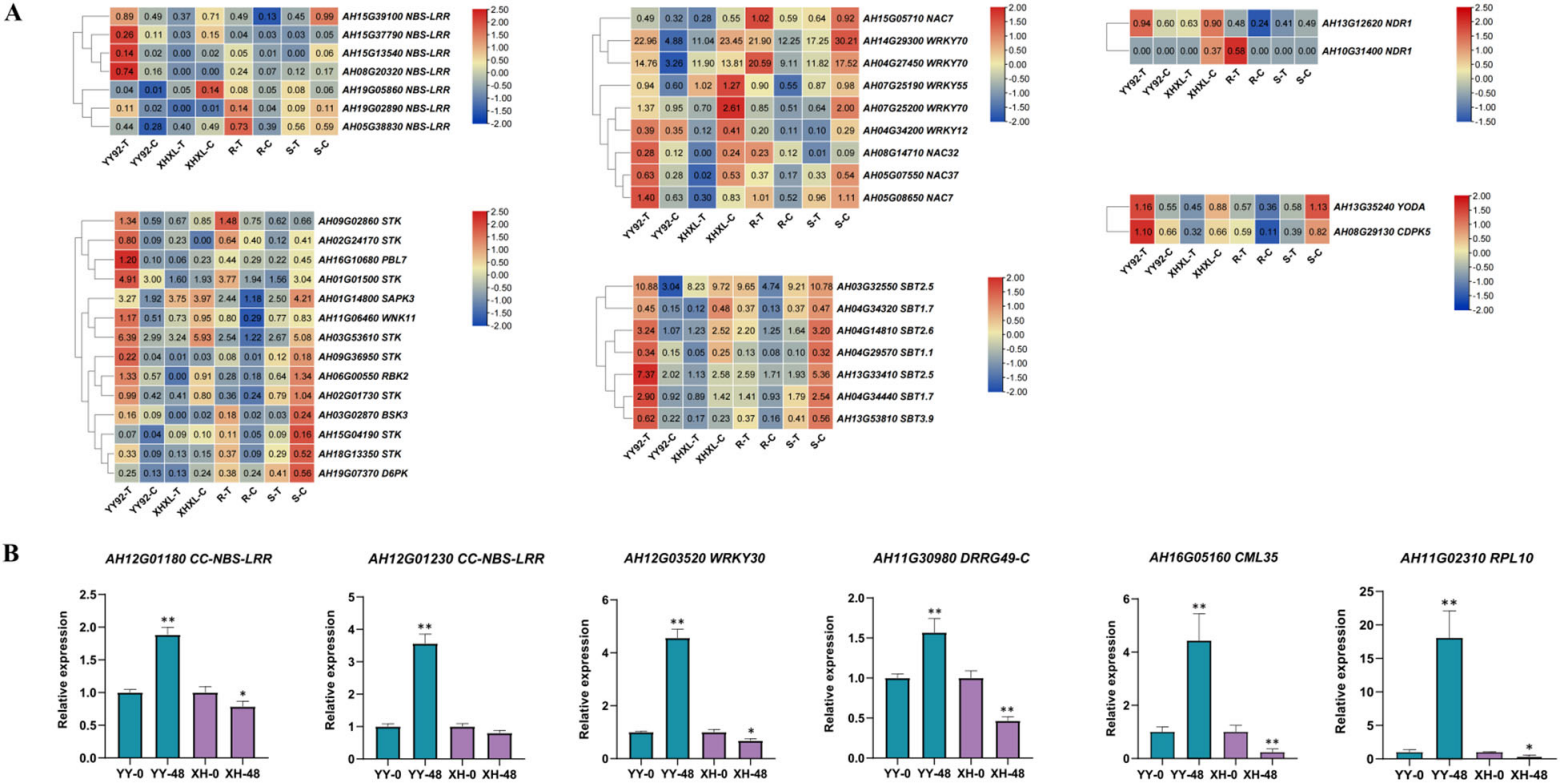
Differentially expressed genes in response to *Rs* stress. **(A)** Relative expression changes of important differential expression genes response to BW stress. A series of enzymes involved in signal transduction pathways and transcription factors related to plant disease resistance. These enzymes include genes associated with disease resistance, serine/threonine-protein kinase, NDR1genes, Ca²^+^ signaling pathway genes, MAPK pathway genes, WRKY transcription factors, NAC transcription factors, and subtilisin-like protease. Differentially expressed genes that are significantly upregulated in YY92 and significantly downregulated in XHXL. The expression level was calculated using log2 (FPKM + 1). Red indicates up-regulated genes, blue indicates down-regulated genes and yellow indicates no expression data. **(B)** Comparative expression analysis of 6 key genes between YY92 (blue) and XHXL (purple) at 48h post *Rs*-inoculation by RT-qPCR. The 2^-ΔΔCt^ method was used for quantification. * and ** indicate significant differences at 0.05 and 0.01 using t-tests, respectively.

Notably, a set of disease resistance signaling pathway-associated genes were significantly upregulated in resistant cultivars but were downregulated or unchanged in susceptible cultivars (**Figure 3A**, **Supplementary Table 19**). The identified genes includes six ETI-related NBS-LRR genes (*AH15G13540, AH19G02890, AH08G20320*), two PTI-associated NDR1 genes (*AH13G12620, AH10G31400*), thirteen serine/threonine-protein kinases (*AH02G24170, AH16G10680, AH09G36950, AH18G13350, AH06G00550, AH02G01730, AH11G06460, AH03G53610, AH19G07370, AH01G14800, AH03G02870, AH15G04190, AH01G01500*); one MAPK pathway gene YODA (*AH13G35240*); one Ca²^+^ signaling gene CDPK5 (*AH08G29130*); five WRKY transcription factors (*AH14G29300, AH04G27450, AH07G25190, AH07G25200, AH04G34200*), four NAC transcription factors (*AH05G07550, AH08G14710, AH05G08650, AH15G05710*); and seven subtilisin-like proteases (*AH13G33410, AH03G32550, AH04G34440, AH04G14810, AH04G34320, AH13G53810, AH04G29570*). qRT-PCR validation of the six key DEGs confirmed expression patterns consistent with RNA-seq data (**Figure 3B**; **Supplementary Table 20**).

### 3.5 Cloning, expression and subcellular location of *AhRRS6*

**Figure 4:**
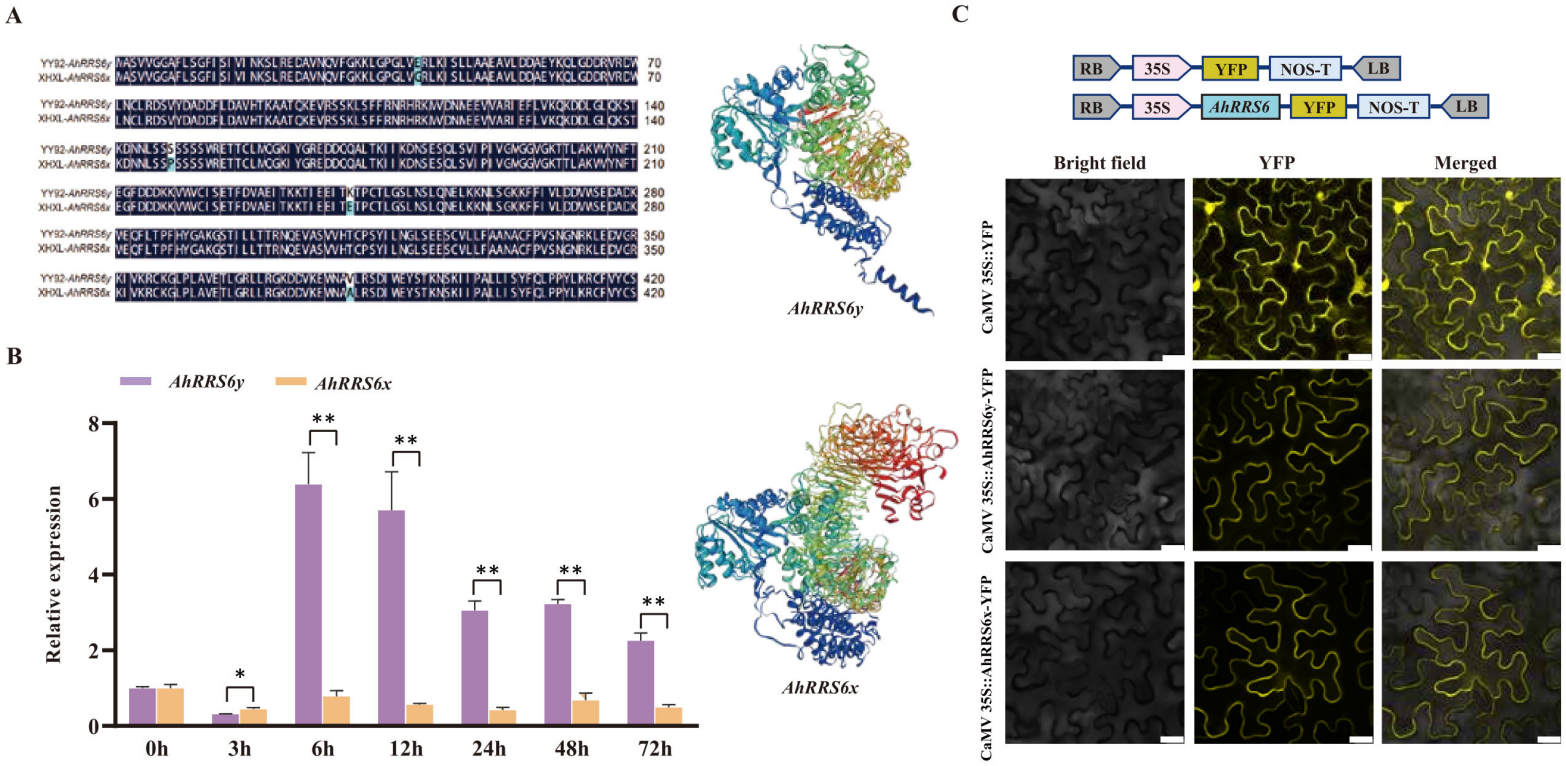
Cloning, expression, and subcellular localization of *AhRRS6*. **(A)** Nucleotide differences in the *AhRRS6* gene between the YY92 and XHXL varieties. **(B)** qRT-PCR analysis of *AhRRS6y* and *AhRRS6x* allele expression levels in peanut (*Arachis hypogaea*) at 0, 3, 6, 12, 24, 48, and 72 hours post-inoculation with *Rs*. The 2^-ΔΔCt^ method was used for quantification. * and ** indicate significant differences at 0.05 and 0.01 using t-tests, respectively. **(C)** Subcellular localization of the *AhRRS6* gene. Observation results of the *AhRRS6y* and *AhRRS6x* genes under a confocal laser scanning microscope, Bar = 25μm.

Based on marker validation and sequence differences between the parents, the candidate gene AH12G01230 was identified and cloned from both the YY92 and XHXL parents (**Figure 2E**; **Figure 3B**; **Supplementary Table S14**), and was named *AhRRS6. AhRRS6* is a CC-NBS-LRR (CNL) gene, comprising a 3,744 bp coding sequence (CDS) with no introns. It encoded a protein containing a coiled-coil (CC) domain, a nucleotide-binding site (NBS) domain, and two leucine-rich repeat (LRR) regions. We designated the allele from the resistant cultivar as *AhRRS6y* and the allele from the susceptible cultivar as *AhRRS6x*. Sequence analysis identified six SNPs between the two alleles that led to four amino acid substitutions within the CC and NBS-ARC domains (not the LRR domain), which in turn induced structural diversity (**Figure 4A**). Tissue-specific expression profiling revealed that *AhRRS6* exhibited highest expression in stem, moderate in stem tip and leaves, and minimal in embryos (**Supplementary Table 21**).

Under *Rs* stress, *AhRRS6y* expression was significantly upregulated compared to *AhRRS6x* at 6 h (peaking at this time point) and remained persistently higher until 72 h, while *AhRRS6x* showed no significant change, suggesting that the *AhRRS6y* allele positively responds to *Rs* stress through its upregulated expression (**Figure 4B**). To determine the sub-cellular localization of *AhRRS6*, we generated AhRRS6y-YFP and AhRRS6x-YFP fusions proteins under the CaMV 35S promoter were transiently expressed in *Nb* leaves. The results showed that AhRRS6y-YFP and AhRRS6x-YFP localized to both plasma membrane and cytoplasm, whereas YFP alone was distributed across multiple subcellular compartments, indicating that *AhRRS6* functions at the plasma membrane and cytoplasm and that SNPs between the alleles do not affect subcellular localization (**Figure 4C**).

### 3.6 Overexpression of *AhRRS6* enhanced resistance to BW in *Nicotiana benthamiana* and *Arabidopsis thaliana*

**Figure 5:**
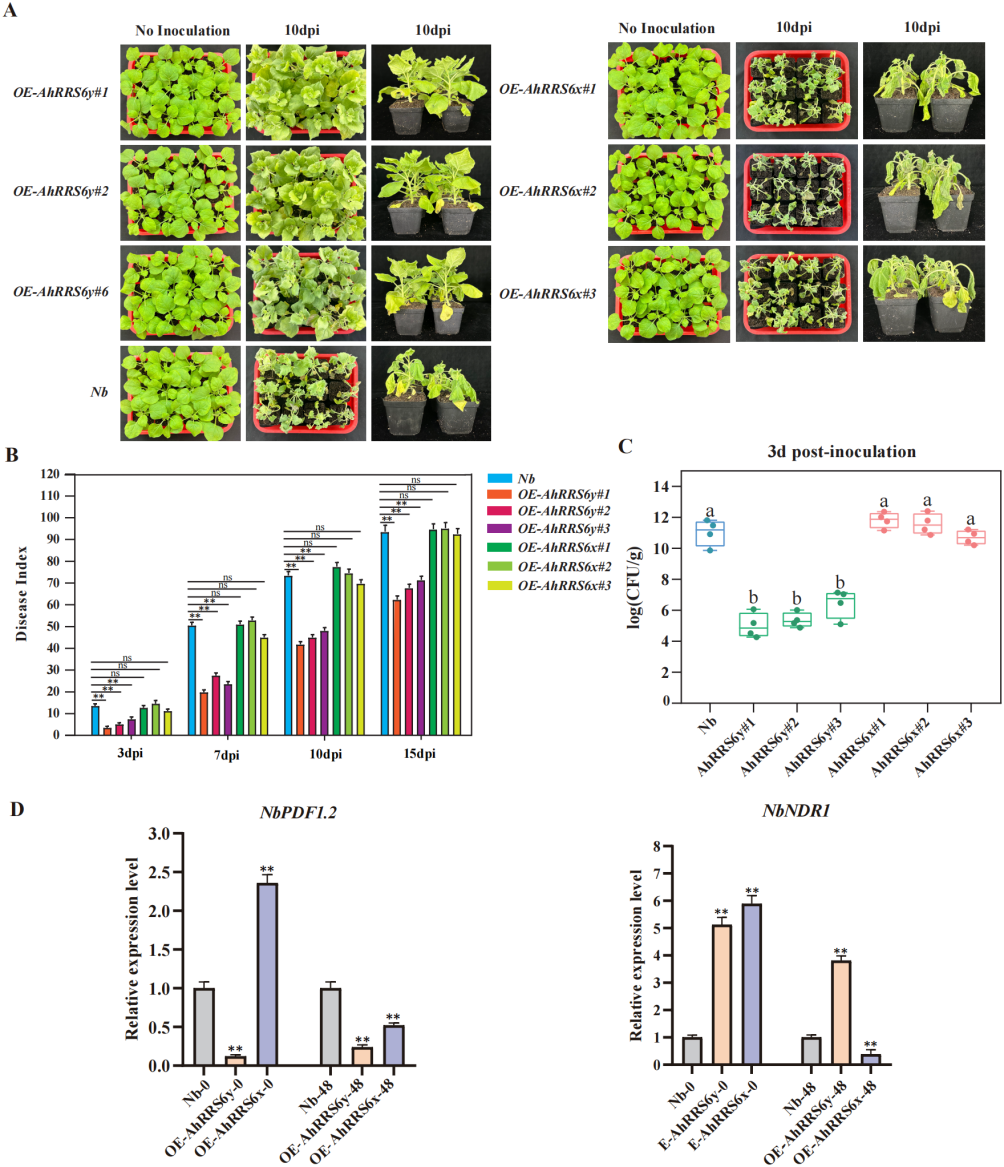
Resistance evaluation of *AhRRS6* transgenic tobacco plants. **(A)** Phenotypes of *AhRRS6y*- and *AhRRS6x*-overexpressing tobacco lines at 10 days post-inoculation (dpi) with *Rs.***(B)** Disease index of transgenic lines following *Rs* inoculation. **(C)** Bacterial titers in transgenic plants at 3 dpi (n = 4; Student’s t-test, P< 0.01). **(D)** Relative expression of *NbPDF1.2* and *NbNDR1* in *AhRRS6y*- and *AhRRS6x*-overexpressing lines at 48 hpi, quantified by qRT-PCR. The 2^-ΔΔCt^ method was used for quantification. * and ** indicate significant differences at 0.05 and 0.01 using t-tests, respectively.

To investigate whether the *AhRRS6* gene confers resistance to BW, we generated transgenic *Nb* (tobacco) lines overexpressing either *AhRRS6y* (resistant allele) or *AhRRS6x* (susceptible allele). Three independent overexpression lines for each allele were developed and advance to the T_3_ generation for phenotypic analysis (**Figure 5A**, **Supplementary Figure 4**). Wild-type *Nb* served as a non-transgenic control. Following inoculation with *Rs*, disease symptoms were quantified using the DI. At 0 dpi with *Rs*, all plants exhibited uniform growth with no significant differences. At day 3 post-inoculation, the DI of *Nb* was 13.49% (**Figure 5B**, **Supplementary Table 22**). The *AhRRS6x* transgenic lines showed slightly higher DI (11.11-14.55%) compared to *AhRRS6y* lines, which maintained a significantly lower average DI of 5.29%. At 7 dpi, the DI in *AhRRS6y* lines ranged from 19.84% to 27.51%, significantly lower than that of both WT (50.52%) and *AhRRS6x* lines (44.97–52.91%). Similarly, the disease index (DI) was consistently and significantly lower in the *AhRRS6y* lines compared to both the WT and *AhRRS6x* lines at day 10 and day 15.

We also transformed *AhRRS6y* and *AhRRS6x* into wild-type *Arabidopsis thaliana* Col-0 plants. Three independent overexpression lines were obtained for each allele. At day 0, all plants exhibited uniform growth with no observable differences. At 3 dpi, all *AhRRS6y* transgenic lines (#1, #2, #3) and Col-0 exhibited no disease symptoms (DI = 0), while the *AhRRS6x* lines showed early wilting (**Supplementary Figures 5A, B**). By 13 dpi, nearly all Col-0 and *AhRRS6x* plants reached 100% mortality, while *AhRRS6y* lines had an average DI of only 55.95% (**Supplementary Table 23**). Bacterial enumeration confirmed that *AhRRS6y*-expressing lines had significantly lower colony-forming units (CFU/g) than Col-0 and *AhRRS6x* lines (**Supplementary Figure 5C**). These results indicated that overexpression of *AhRRS6y* delays disease progression and significantly enhanced resistance to BW in both *Nb* and *Arabidopsis*. In contrast, *AhRRS6x* did not confer protection despite differing from *AhRRS6y* by only a few nucleotide substitutions.

To gain insight into the underlying defense mechanisms, we assessed the expression of known defense-related marker genes in the transgenic *Nb* lines by qRT-PCR following *Rs* inoculation. Key genes including *NbHSR203J*, *NbMEK2*, and *NbEDS1* were strongly upregulated in *AhRRS6y* lines, especially under pathogen stress (**Supplementary Figure 6**). *AhRRS6y* overexpression downregulated *NbPDF1.2* expression in *Nb*, whereas *AhRRS6x* overexpression enhanced its expression; however, both lines showed significant *NbPDF1.2* downregulation post-inoculation (**Figure 5D**). Notably, both alleles induced *NbNDR1* under basal conditions, but after infection, *NbNDR1* expression stayed high in *AhRRS6y* lines but decreased in *AhRRS6x* lines.

### 3.7 Effects of *Rs* infection on malondialdehyde content and ascorbate peroxidase activity in *AhRRS6*-transgenic tobacco

**Figure 6:**
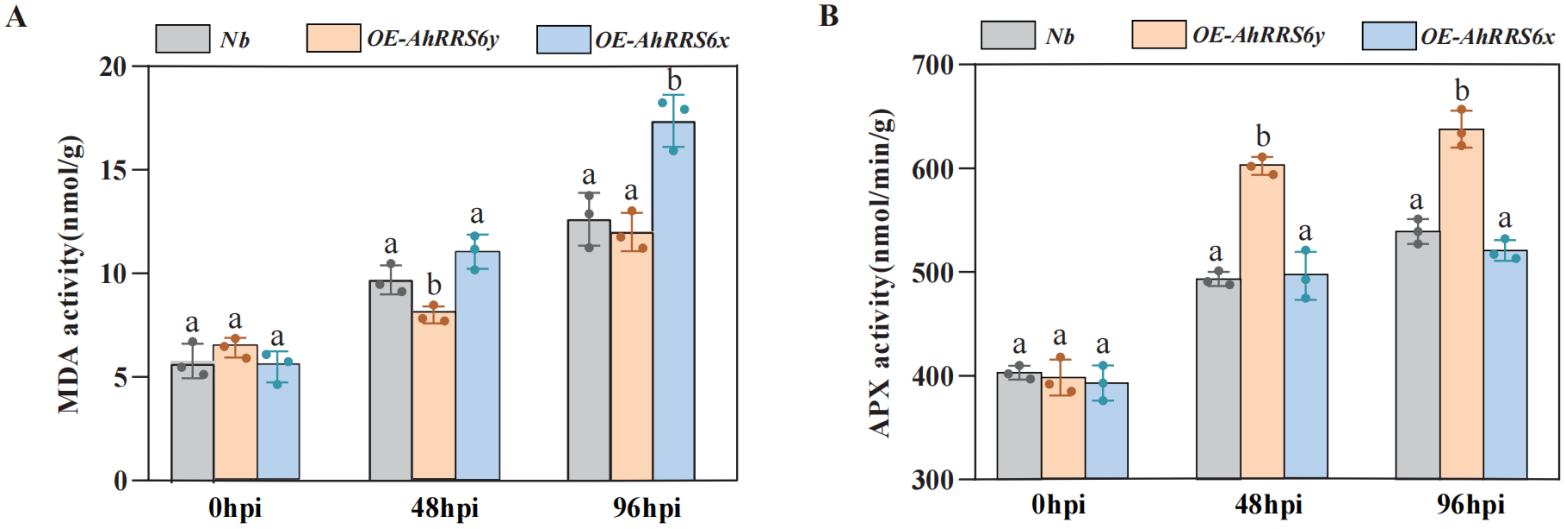
Determination of MDA content and APX activity in *AhRRS6* transgenic tobacco under *Rs* stress. **(A)** Malondialdehyde (MDA) content in *AhRRS6* transgenic lines. **(B)** Ascorbate peroxidase (APX) activity in *AhRRS6* transgenic lines (n = 3; Student’s t-test, P< 0.01).

To assess the impact of Rs infection on oxidative damage and antioxidant defense, we measured malondialdehyde (MDA) content and ascorbate peroxidase (APX) activity in wild-type (*Nb*) and overexpression lines (OE-*AhRRS6y*, OE-*AhRRS6x*) at 0, 48, and 96 hours post-inoculation (hpi). At 0 hpi, MDA dynamics (**Figure 6A**) revealed no significant baseline differences among lines, indicating that transformation did not affect basal oxidative status. By 48 hpi, MDA increased significantly in all lines, with Nb and OE-*AhRRS6x* (11.02 ± 0.83 nmol/g FW) exceeding OE-*AhRRS6y*. At 96 hpi, OE-*AhRRS6y* maintained the lowest MDA (12.00 ± 0.92 nmol/g FW), whereas OE-*AhRRS6x* peaked at 17.33 ± 1.26 nmol/g FW – significantly higher than both *Nb* and OE-*AhRRS6y*.

Concurrently, APX activity (**Figure 6B**) showed consistent baseline levels at 0 hpi, confirming unchanged basal antioxidant capacity. Pathogen infection induced APX upregulation by 48 hpi, most notably in OE-*AhRRS6y* (602.33 ± 8.51 μmol min^-^¹ g^-^¹ FW; 1.51-fold increase), exceeding *Nb* (493.33 ± 6.81) and OE-*AhRRS6x* (496.33 ± 23.18) by 109.00 and 106.33 μmol min^-^¹ g^-^¹ FW, respectively. This elevated activity persisted in OE-*AhRRS6y* at 96 hpi (peak: 637.67 ± 14.52 μmol min^-^¹ g^-^¹ FW), while OE-*AhRRS6x* showed a weaker response similar to *Nb*. Collectively, the suppressed MDA accumulation and sustained APX induction in OE-*AhRRS6y* indicate enhanced ROS scavenging that preserves membrane integrity and contributes to disease resistance, contrasting with the limited antioxidant response in OE-*AhRRS6x*.

## 4 Discussion

### 4.1 BSR-seq was an effective way for directly mapping candidate gene response to bacterial wilt

In this study, we demonstrated that BSR-seq represents an efficient and economical method for high-resolution mapping of candidate genes associated with BWR in peanut. This approach overcame the challenges of the allotetraploid peanut genome and facilitated functional insights into defense responses against Rs. Given the complexity of the allotetraploid peanut genomic background and low recombination rate of beneficial loci, which poses challenges in studying their genetic traits (Zhuang et al., 2019). This study aimed to decipher the genetic underpinnings of BWR in peanut, a major constraint in peanut production caused by *Rs.* To screen for BWR genes, BSR-Seq was employed. BSR-Seq has been widely used for rapid QTL mapping and candidate gene discovery in crops such as soybean (Huang et al., 2024) and wheat (Saxesena et al., 2022). BSR-Seq enabled high-resolution QTL mapping and directly cloning of candidate expressed genes. However, its application to peanuts had remained limited. Peanut resistance to bacterial wilt was influenced by complex interplay of environmental factors. Traditionally, the evaluation of disease resistance in nurseries has relied on measuring survival rates and has proven instrumental in mapping QTLs for bacterial wilt resistance (Luo et al., 2019; Zhang et al., 2022). However, it was prone to interference from factors such as temperature fluctuations, soil conditions, and human error. In our previous studies, we developed an artificial inoculation technique for peanuts to address these challenges. This technique involved leaf cutting, followed by infection with *Rs* and subsequent assessment of resistance using a DI (Zhao et al., 2016; Zhang et al., 2017). This enabled the identification of relevant genomic regions using the BSR-seq method.

**Figure 7:**
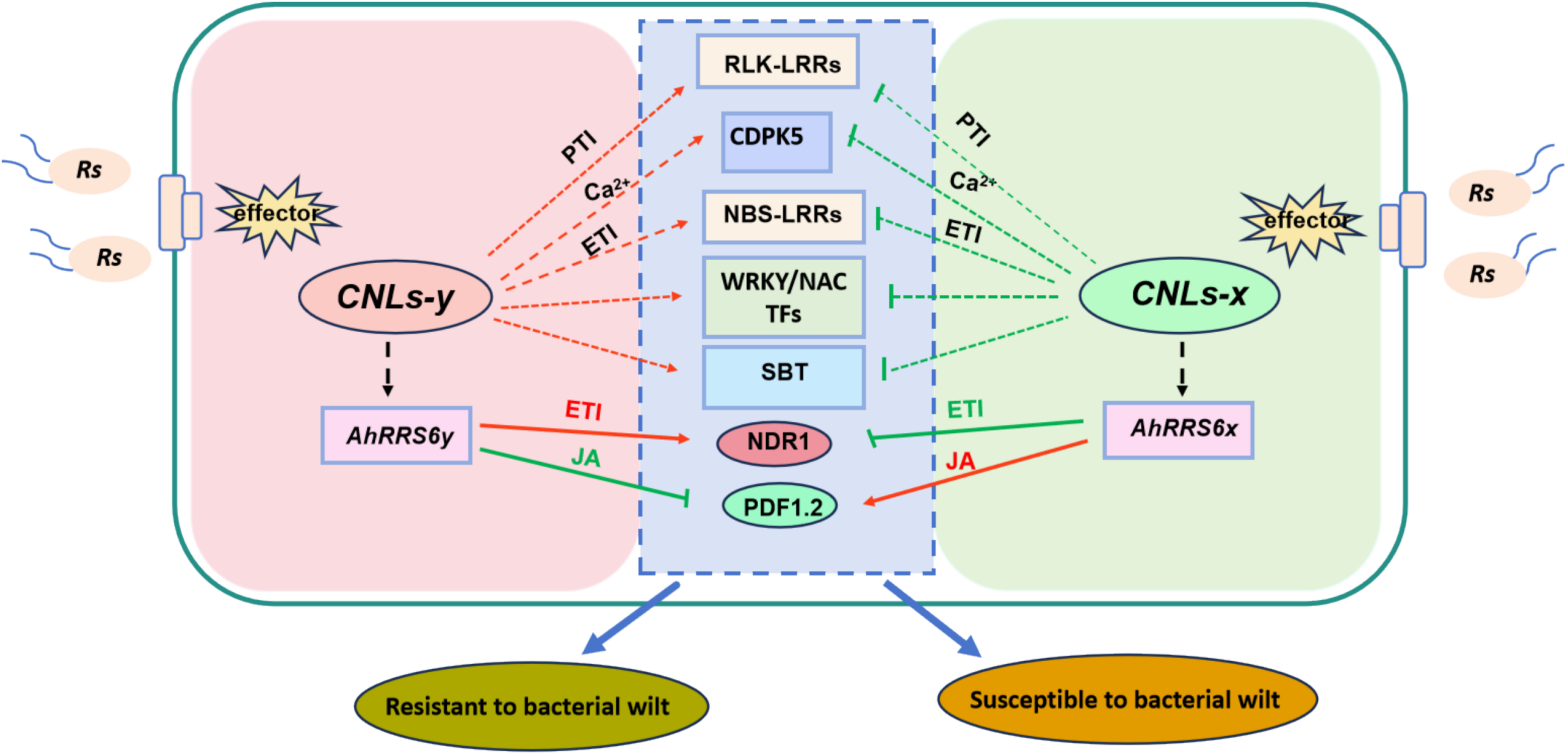
Predicted schematic model of peanut response to *Rs* infection. *CC-NBS-LRR (CNL)* genes, as key genes within the mapped region, play a critical role in peanut resistance to bacterial wilt. The *AhRRS6y* allele (from resistant cultivar YY92) was upregulated under pathogen stress and positively regulated *NBS-LRRs* genes, *serine/threonine-protein kinase, NDR1genes, calcium metabolism (CDPK5), WRKY transcription factors (WRKY12, WRKY55, WRKY70), NAC transcription factors (NAC7, NAC32, NAC37)*, and *subtilisin-like protease (SBT1.1, SBT1.7, SBT2.5, SBT3.9)* based on BSR-seq; its overexpression activated *NbNDR1* but suppressed *NbPDF1.2* (JA pathway) per qRT-PCR. Conversely, the *AhRRS6x* allele (from susceptible cultivar XHXL) was downregulated and negatively regulated the same gene sets, while its overexpression enhanced *NbPDF1.2* but repressed *NbNDR1*. Red arrows denote gene upregulation; green T-bars indicate downregulation.

In our study, we employed BSR-Seq and identified 33,877 and 36,158 high-quality and SNP markers before and after inoculation with *Rs* (**Supplementary Tables 3** and **4**). The observed variation likely reflects *Rs*-induced transcriptional changes. From this study, we identified 187 related genes associated with BWR, including 5 NBS-LRR genes which were considered as key candidates (Zhang et al., 2017). Furthermore, a set of important DEGs related to peanut resistance to BW were also identified, and some genes such as *WRKY70, SBT2.5* were further confirmed through qRT-PCR (**Figure 3E**). These genes are involved in key immune pathways including the hypersensitive response (HR), MAPK signaling, and calcium signaling, forming the foundation for a working model of peanut–*Rs* interaction. Based on these findings, we proposed a potential molecular network illustrating the interaction between peanut and *Rs* (**Figure 7**). Building on the BSR-seq analysis, we identified two distinct QTL intervals on chromosome 12 that significantly contribute to BWR. To confirm and refine these regions, we integrated the transcriptomic data with QTL mapping, allele-specific SNP validation, and gene annotation to pinpoint functionally relevant loci. Finally, we positionally cloned a *CC-NBS-LRR* gene named *AhRRS6* (**Figure 5**) and obtained three gene-specific SNP Marks validated in a panel of 10 resistant and 10 susceptible peanut varieties for breeding deployment.

### 4.2 A novel region was fine-mapped on Chr12 for bacterial wilt resistance

Previously, we identified two major QTLs *(qBW-1 and qBW-2)* on linkage groups LG1 and LG10, respectively, using traditional genetic mapping techniques in F_2_ plants, based on RAD- and BSA-seq analysis. In an F_8_ RIL population, a single QTL linked to three peaks on LG1, located on B02 in the diploid peanut *Arachis ipaensis* (Zhao et al., 2016). Later, a QTL named *qBWRB02.1*, spanning a potential 5.14 Mb interval on chromosome B02, was identified (Luo et al., 2020). Within the confidence interval of *qBWRB02.1*, two adjacent genomic regions (2.81–4.24 Mb and 6.54–8.75 Mb) were further delineated and designated as *qBWRB02.1–1* and *qBWRB02.1-2*, based on two diploid reference genomes. Additionally, 22 NBS-LRR genes with nonsynonymous mutations in the 7.2 Mb region on chromosome 12 (Chr12) were reported to be significantly associated with the resistance to *Rs* (Zhang et al., 2022). In our study, we mapped two candidate genomic regions on Chr12 associated with BWR, spanning 1.11 Mb (1,763,660–2,877,695 bp) and 1.03 Mb (3,665,856–4,691,788 bp), identified through association analysis using ED and ΔSNP-index methods based on BSR-seq. Using QTL mapping based on an existing genetic map (Zhuang et al., 2019), a region was identified on linkage group 12 (2847722bp–6381141bp), which validated the reliability of the 3,665,856–4,691,788 bp interval, while the 1,763,660–2,877,695 bp interval was newly identified in this study.

We fine-mapped and selected five expressed NBS-LRR genes with nonsynonymous SNPs as candidate genes, including *AH12G01180* and *AH12G01230*, located within a 0.6 Mb interval. Two nonsynonymous mutations in *AH12G01230* (Chr12–1931823 and Chr12-1932270) were absent in susceptible progeny, warranting further study. Functional analysis of its alleles from resistant and susceptible genotypes provided strong evidence for its role in modulating peanut immunity to *Rs*. Further analysis of *R* genes types and classifications on this regions revealed that most of these genes belong to the *CC-NBS-LRR* class. Approximately 25% of *NLR* genes exist as ‘genetic singletons’ within plant genomes, while the remainder often forms genetic clusters near the telomeres (Jacob et al., 2013). The Clustering of the same class of *R* genes may facilitate mutation generation, providing genetic diversity for resistance evolution in response to pathogens (Jacob et al., 2013; Kourelis et al., 2021). Based on this, we hypothesize that this region is a genetic hotspot for cultivated peanut resistance. This region likely coevolved with *Rs* pathogens, with different *R* genes contributing diverse effects to overall resistance observed in various peanut varieties.

### 4.3 *AhRRS6* conferred resistance to bacterial wilt in heterozygous plant

*AhRRS6* was identified as typical CC-NBS-LRR-type of protein (**Figure 2E**). Comparison of *AhRRS6* homologs from resistant and susceptible peanut parents revealed 6 SNPs, resulting in 4 amino acid substitutions that altered protein conformation and function. Expression pattern showed differential expression, with *AhRRS6y* exhibiting higher expression levels in response to *Rs* (**Figure 4B**), consistent with its functional diversity in resistance. Transcriptome profiling indicated that *AhRRS6* was predominantly expressed in the stem and roots, with only trace expression in the embryo and pericarp. This pattern aligned with the established knowledge that *Rs* infects plants through the roots by colonizing the vascular bundles (Peeters et al., 2013).

Subcellular localization studies using the *AhRRS6*::YFP fusion protein in *N. benthamiana* leaf cells showed that the *AhRRS6* protein was localized to the plasma membrane and cytoplasm (**Figure 4C**). This observation was consistent with reports that R genes typically function in the cytoplasm (Dodds and Rathjen, 2010). However, NLR proteins have also been shown to localize to diverse subcellular compartments, including the cytoplasm, nucleus, plasma membrane (PM), vacuolar membrane, and endoplasmic reticulum (Cesari, 2018). The observed localization of *AhRRS6* may be influenced by structure changes or protein modifications following interaction with target gene. Similar mechanisms have been reported in *Arabidopsis*, where the RRS1-R protein translocated to the nucleus after interacting with the effector PopP2 (Le Roux et al., 2015; Sarris et al., 2015). This dynamic localization pattern of *AhRRS6* supports its potential role in peanut resistance to *Rs*.

Transgenic evaluation in heterozygous *Nb* and *Arabidopsis* demonstrated that overexpression of *AhRRS6y* significantly enhanced resistance to BW resulting in a much lower disease index (**Figure 5**, **Supplementary Figure 5**). In contrast, *AhRRS6x* from the susceptible XHXL exhibited high susceptibility, similar to that of non-transgenic control plants. Under biotic stress, elevated energy states of electrons in plant cells drive excessive reactive oxygen species (ROS) formation. These highly oxidizing compounds induce oxidative damage to proteins and membrane lipids, compromising cellular integrity. Malondialdehyde (MDA), a terminal lipid peroxidation product, serves as a robust biomarker for pathogen-induced plasma membrane damage (Mittler et al., 2022). Following *Rs* inoculation, MDA levels significantly increased in all lines (*Nb*, *AhRRS6y*-overexpressing, and *AhRRS6x*-overexpressing) compared to the 0-hour baseline. Notably, the *AhRRS6x*-overexpressing line exhibited the most pronounced elevation in MDA content. This indicates that pathogen-induced ROS accumulation triggered severe lipid peroxidation. In *AhRRS6x*-overexpressing plants, suppressed defense responses, likely caused uncontrolled ROS bursts leading to excessive MDA accumulation, reflecting pronounced oxidative damage and cell death (Waszczak et al., 2018). Ascorbate peroxidase (APX) is a key enzyme in ROS detoxification, and is crucial for mitigating oxidative stress during pathogen infection (Hasanuzzaman et al., 2020). At 48–96 hours post-inoculation, APX activity was significantly upregulated in the *AhRRS6y*-overexpressing line compared to the *Nb* control and *AhRRS6x*-overexpressing line. This enhanced APX activity demonstrates that *AhRRS6y*-overexpressing plants bolster their antioxidant capacity via an APX-mediated H_2_O_2_ scavenging system, maintaining cellular redox homeostasis and enhancing resistance against pathogen invasion (Foyer and Hanke, 2022).

The diversity in resistance was likely explained by the four amino acid variations between the parental lines, which affect the CC and NB-ARC regions of *AhRRS6*. These regions had been shown to play critical roles in the specific resistance functions of R-genes (Sun et al., 2020). This genetic basis for resistance differences between the two varieties highlights the significance of these amino acid changes. Despite the broad host range of *Rs* only two resistant QTLs have been reported in *Arabidopsis* via positional cloning: one encoding the TIR-NBS-LRR (*RRS1-R)* and the receptor like protein kinase *(ERECTA)*. The resistance functions and underlying mechanisms of these genes have been well-characterized (Deslandes et al., 2002; Godiard et al., 2003). In peanut, *AhRRS5* encoding an NBS-LRR protein and the *AhRLK1* coding a receptor like kinase were cloned through transcriptome analysis and shown to confer resistance to BW (Zhang et al., 2017, 2019). Importantly, *AhRRS6* was the first reported *CC-NBS-LRR* resistance gene obtained by positional cloning in crops, demonstrating resistance to diverse *Rs* in plants. These findings underscored the significance of *AhRRS6* in understanding and improving resistance to BW in peanut and other crops.

### 4.4 Peanut’s resistance to bacterial wilt involving multiple metabolic pathway

In this study, we identified several key genes associated with plant immune signaling pathways (**Figure 3A**). Notably, these genes exhibited contrasting expression patterns under *Rs* stress: up-regulation in resistant peanut varieties compared to down-regulation in susceptible ones. Functional validation via qRT-PCR confirmed that several of these genes positively regulate peanut resistance (**Figure 3B**). Among these are NBS-LRR genes, encoding intracellular immune receptors that recognize pathogen effector proteins to activate Effector-Triggered Immunity (ETI), often culminating in a hypersensitive response (HR), a well-established defense mechanism in plants (Jones and Dangl, 2006; Dodds and Rathjen, 2010). Additionally, Receptor-Like Kinases (RLKs) function as cell surface Serine/Threonine Kinases (STKs), directly sensing Pathogen-Associated Molecular Patterns (PAMPs) during Pattern-Triggered Immunity (PTI) or host targets during ETI, and transducing signals intracellularly via phosphorylation (Yuan et al., 2021). Downstream signaling components identified include genes involved in the calcium signaling pathway, such as CDPK5, which encodes calcium sensor proteins known to enhance salicylic acid (SA)-mediated resistance against bacterial pathogens, as demonstrated in *Arabidopsis* (Dubiella et al., 2013). Similarly, genes within the MAPK signaling pathway (e.g., YODA) were implicated in enhance resistance to soil-borne fungal pathogens like Verticillium wilt by activating modules like MKK4-MPK6 and inducing pathogenesis-related (PR) genes including chitinases (Meng et al., 2015). Furthermore, transcription factors played significant roles: WRKY factors (e.g., OsWRKY70 in rice regulating stomatal closure for bacterial resistance (Shimono et al., 2007); NtWRKY55 in *Nb* whose silencing increased susceptibility (Wang et al., 2015) and NAC transcription factors (e.g., GhNAC7 in cotton conferring resistance to Verticillium dahlia (Xu et al., 2024). Collectively, our results highlight the critical involvement of genes mediating ETI, PTI, MAPK signaling, Ca²^+^ signaling, and the regulatory functions of WRKY and NAC transcription factors in peanut’s defense response against *Rs.* Specifically, five NBS-LRR genes were mapped to the BWR regions using BSR-seq. Among these, the CNL gene *AhRRS6* significantly enhanced resistance in transgenic tobacco plants, while the corresponding allelic gene from the susceptible parent did not confer resistance. qRT-PCR analysis revealed that marker genes related to Effector-Triggered Immunity (ETI), such as *NbEDS1*, hyper-response marker gene *HSR203J*, were upregulated in *AhRRS6*-transgenic plants following *Rs* challenge. Similarly, SA signaling pathway marker gene *PR1*, MAPK signaling pathway (*MEK2)*, and PTI were also upregulated. Conversely, *NbPDF1.2*, a marker associated with the jasmonic acid (JA) pathway, was downregulated in *AhRRS6y*-transgenic plants. *NbNDR1*, a key gene mediating ETI responses in CNL-type protein resistance pathway, exhibited upregulated expression in *AhRRS6y*-transgenic plants inoculated with *Rs*, while it was downregulated in *AhRRS6x*-transgenic plants. These findings confirmed that peanut resistance to BWR involves multiple immune signaling pathways as evidenced by differential regulation of key marker genes in response to Rs (**Figure 7**). Collectively, these findings highlight *AhRRS6* as a key resistance gene and support a model in which CC-NBS-LRR proteins orchestrate multiple signaling cascades, including ETI, PTI, MAPK, and SA pathways, to confer durable resistance to BW in peanut.

## 5 Conclusion

This study employed a high-generation recombinant inbred line (RIL) population (581) from resistant and susceptible parents, identified resistant and susceptible progeny, and constructed pooled samples. BSR-seq identified two QTL loci on chromosome 12 (1.11 Mb and 1.03 Mb), along with 11 nonsynonymous SNPs in five NBS-LRR genes, three of which were validated in the progeny. QTL-seq confirmed that the region at 1.11 Mb (1,763,660–2,877,695 bp) on chromosome 12 was a novel locus. *AhRRS6* was identified as a candidate gene, with overexpression of *AhRRS6y* in *Nicotiana benthamiana* and *Arabidopsis* enhancing resistance, while overexpression of *AhRRS6x* increased susceptibility. Overexpression of *AhRRS6* also activated key genes in the HR, PTI, MAPK, ETI, and JA signaling pathways. Differential gene expression analysis indicated that peanut responses to *Rs* involve PTI, ETI, MAPK, Ca^2+^, and other pathways. Transgenic tobacco expressing *AhRRS6y* exhibited lower malondialdehyde (MDA) content and higher ascorbate peroxidase (APX) activity during the early stage of *Rs* infection compared to *AhRRS6x*-overexpressing lines. Future research will investigate the molecular mechanisms underlying *AhRRS6*-mediated resistance. Overall, this study provides deeper insights into bacterial wilt resistance in peanuts, identifies key genes and molecular markers for breeding resistant cultivars, and unveils the potential regulatory network controlling resistance to *Rs.*

## Data Availability

The raw sequence data reported in this paper have been deposited in the Genome Sequence Archive (Genomics, Proteomics & Bioinformatics 2025) of the National Genomics Data Center (Nucleic Acids Res 2025), China National Center for Bioinformation/Beijing Institute of Genomics, Chinese Academy of Sciences (accession number: GSA: CRA037312), and are publicly accessible at https://ngdc.cncb.ac.cn/gsa.

## Supplementary material

Supplementary Figure 1 A schematic diagram illustrating the process of aligning reads from bulk samples to the reference genome, performing variant detection, calculating the SNP-index for variant sites within the bulk samples, and determining the ΔSNP-index between resistant and susceptible bulks. Both the treatment group and the control group follow the calculation method described above.

Supplementary Figure 2 Locating candidate intervals for peanut resistance to bacterial wilt disease based on control and treatment group data. **a.** Chromosomes of cultivated tetraploid peanut (*Arachis hypogaea* subsp. *fastigiata* var. *vulgaris*) cultivar ‘Shitouqi’reference genome; **b.** Genome-wide density of total genes; **c.** High-quality SNP variants in the control group; **d.** Euclidean Distance(ED) values; **e.** ΔSNP-index plot; The shaded area with red arrows represents the loci exceeding the threshold value; The colorful lines in the middle represent homologous gene pairs.

Supplementary Figure 3 Identifying Regions and Genes Associated with Resistance to Bacterial wilt Disease using ΔSNP-index and ED values methods in treatment group. **(A)**. Locating Candidate Regions Associated with Bacterial wilt Disease in Peanuts Using the Delta SNP-Index Method. The X-axis represents the chromosome name. Each point of different colors represents an SNP locus. The black dashed line connects the mean SNP-index values within each window (2Mb windows sliding in 10 kb steps), and the red line represents the threshold line fitted through 1000 iterations to achieve a 90% confidence level. **(B)**. Locating Candidate Regions Associated with Bacterial wilt Disease in Peanuts Using the Euclidean Distance method. The X-axis represents the names of chromosomes, with points of different colors representing Euclidean Distance (ED) values. The black line indicates the mean ED values within each window (2Mb windows sliding in 10 kb steps), while the red line represents the threshold line.

Supplementary Figure 4 Phenotypes of *AhRRS6* transgenic tobacco lines at 10 days post-inoculation with *Rs.* Images show *Nb* control, OE-AhRRS6y, and OE-AhRRS6x lines pre-inoculation and post-inoculation.

Supplementary Figure 5 Resistance evaluation of *AhRRS6* transgenic *Arabidopsis*. **(A).** Phenotypes of Col-0 wild-type, Nd-1, *AhRRS6y*-, and *AhRRS6x*-overexpressing lines at 11 days post-inoculation with *Rs*. **(B)**. Disease index of transgenic lines post-inoculation. **(C).** Bacterial titers in plants at 3 dpi (n = 4; Student’s t-test, P< 0.01).

Supplementary Figure 6 qRT-PCR analysis of key genes in plant disease resistance signaling pathways. Expression levels of defense-related marker genes (*NbPR1, NbEDS1, NbARCE, NbMEK2, NbHSP203J*) in control *Nb* plants and *AhRRS6y/AhRRS6x* transgenic lines, before and after inoculation with *Rs.* Relative expression was calculated using the 2^-ΔΔCt^ method. * and ** denote statistically significant differences at *p*< 0.05 and *p*< 0.01, respectively (Student’s t-test).

Supplementary Figure 7 The structure and evolution of R genes within the first 15 Mb region of chromosome 12.

Supplementary Table 1 Details on the recombinant inbred lines (RILs) selected for construction of resistant and susceptible bulks for BWR in different seasons.

Supplementary Table 2 Data summary of RNA-seq for YY92, XHXL and their derived resistant and susceptible bulks obtained from 30 homozygous resistant and 30 homozygous susceptible F2:13 families before and after bacteria stress respectively.

Supplementary Table 3 High-quality SNP variants in the treatment group. Chr: Chromosome Name; Pos: SNP sites; Gene_id: Gene where the SNP is located; Ref: The SNP type at this locus in the reference genome of the cultivated tetraploid peanut (*Arachis hypogaea* subsp. *fastigiata* var. *vulgaris*) cultivar ‘Shitouqi’; Alt: Mutation type; Depth: Sequencing depth; AlleDp: Allelic Depth; Effect: The annotation information of SNP; Codon_change: Amino acid changes caused by SNP mutations. YY92-T: YY92 parental treatment group; XHXL-T: XHXL parental treatment group; R-T: Resistance mixed pool treatment group; S-T: Susceptible mixed pool treatment group;.

Supplementary Table 4 High-quality SNP variants in the control group. Chr: Chromosome Name; Pos: SNP sites; Gene_id: Gene where the SNP is located; Ref: The SNP type at this locus in the reference genome of the cultivated tetraploid peanut (*Arachis hypogaea* subsp. *fastigiata* var. *vulgaris*) cultivar ‘Shitouqi’; Alt: Mutation type; Depth: Sequencing depth; AlleDp: Allelic Depth; Effect: The annotation information of SNP; Codon_change: Amino acid changes caused by SNP mutations. YY92-C: YY92 parental control group; XHXL-C: XHXL parental control group; R-C: Resistance mixed pool control group; S-C: Susceptible mixed pool control group.

Supplementary Table 5 Calculate the ED value for each window in the control group using the ED algorithm sliding window width.

Supplementary Table 6 Calculate the SNP-index value for each window in the control group using the ΔSNP-index algorithm.

Supplementary Table 7 The candidate intervals of the control group using ED algorithm method and ΔSNP-index method.

Supplementary Table 8 Calculate the ED value for each window in the treatment group using the ED algorithm.

Supplementary Table 9 Calculate the SNP-index value for each window in the treatment group using the ΔSNP-index algorithm.

Supplementary Table 10 The candidate intervals of the treatment group using ED algorithm method and ΔSNP-index method.

Supplementary Table 11 Genes contained within the two QTL loci identified for peanut resistance to bacterial wilt through QTL mapping using the SNP-index and ED algorithms on the experimental and control groups.

Supplementary Table 12 Identification of SNPs between resistant and susceptible bulks in treatment group using QTL-seq approach (ED and ΔSNP-index) for resistance to *Rs* infection.

Supplementary Table 13 Identification of SNPs between resistant and susceptible bulks in control group using QTL-seq approach (ED and ΔSNP-index) for resistance to *Rs* infection.

Supplementary Table 14 Identification of genes between resistant and susceptible bulks using QTL-seq approach (ED and ΔSNP-index) for resistance to *Rs* infection.

Supplementary Table 15 Summary of whole genome re-sequencing of YY92, XHXL and their derived 10 resistant and 10 susceptible springs.

Supplementary Table 16 BSR-seq identified candidate SNP sites consistent with those called by whole-genome resequencing.

Supplementary Table 17 1865 DEGs that consistently showed differential expression between the resistant parents and bulks.

Supplementary Table 18 2711 DEGs that consistently showed differential expression between the susceptible parents and bulks.

Supplementary Table 19 Key plant disease resistance genes: upregulated in resistant lines, downregulated in susceptible lines upon *Rs* induction.

Supplementary Table 20 Primer sequences for qRT-PCR and vector construction.

Supplementary Table 21 The expression levels of *AhRRS6* in different peanut tissues.

Supplementary Table 22 Disease assessment in *AhRRS6* transgenic tobacco plants after *Rs* inoculation.

Supplementary Table 23 Resistance evaluation in *AhRRS6* transgenic *Arabidopsis* at 11 days post-inoculation with *Rs.*
