# Supplementary figures and images for: Positional cloning of a peanut CC-NBS-LRR gene, AhRRS6, confers resistance to Ralstonia solanacearum
Source: Front Plant Sci. 2026 Feb 3;16:1718434. doi: 10.3389/fpls.2025.1718434 (PMC12960648; doi:10.3389/fpls.2025.1718434)

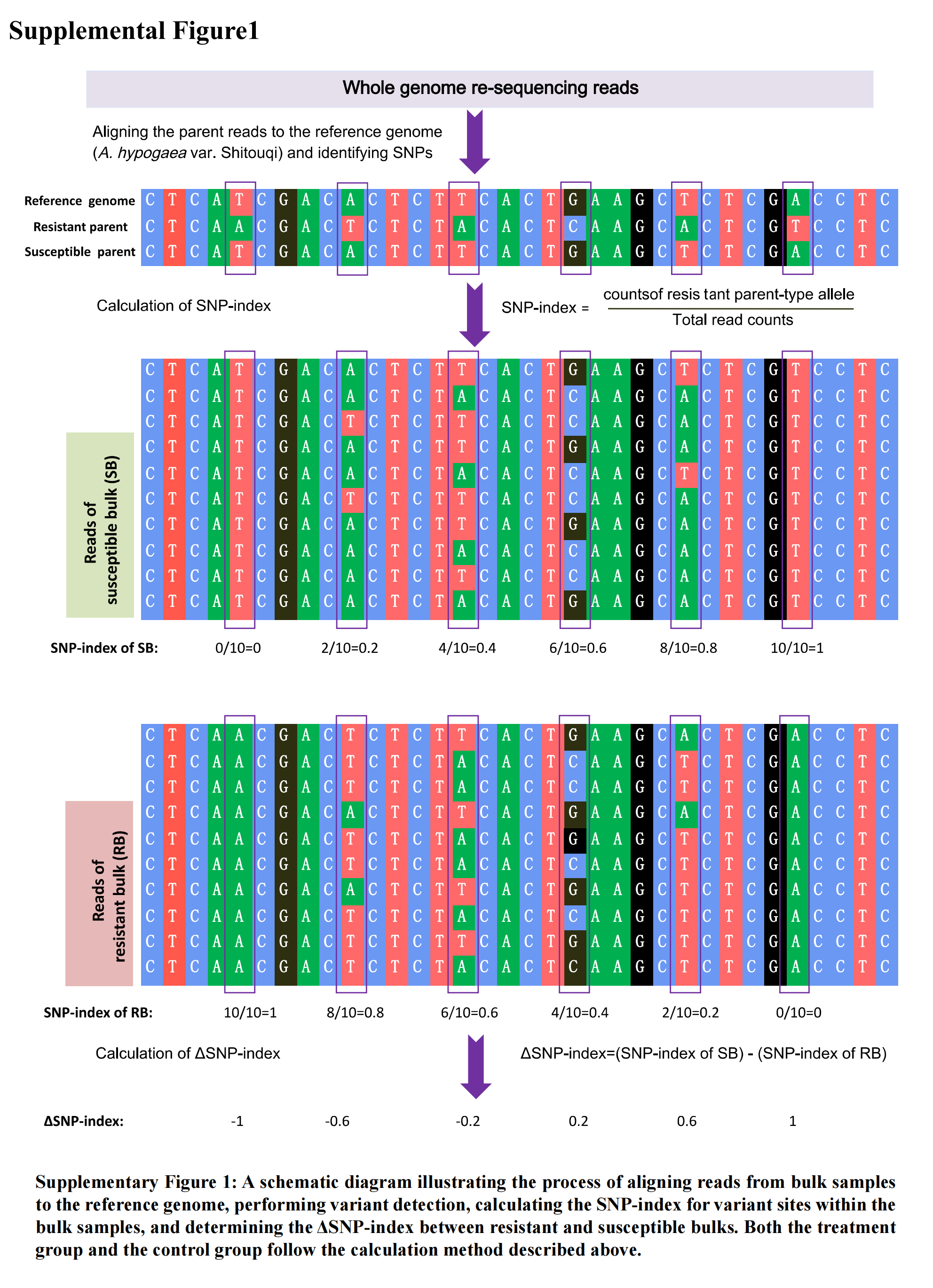

Supplement: Supplementary Figure 1 — A schematic diagram illustrating the process of aligning reads from bulk samples to the reference genome, performing variant detection, calculating the SNP-index for variant sites within the bulk samples, and determining the ΔSNP-index between resistant and susceptible bulks. Both the treatment group and the control group follow the calculation method described above. [file Image1.tif]

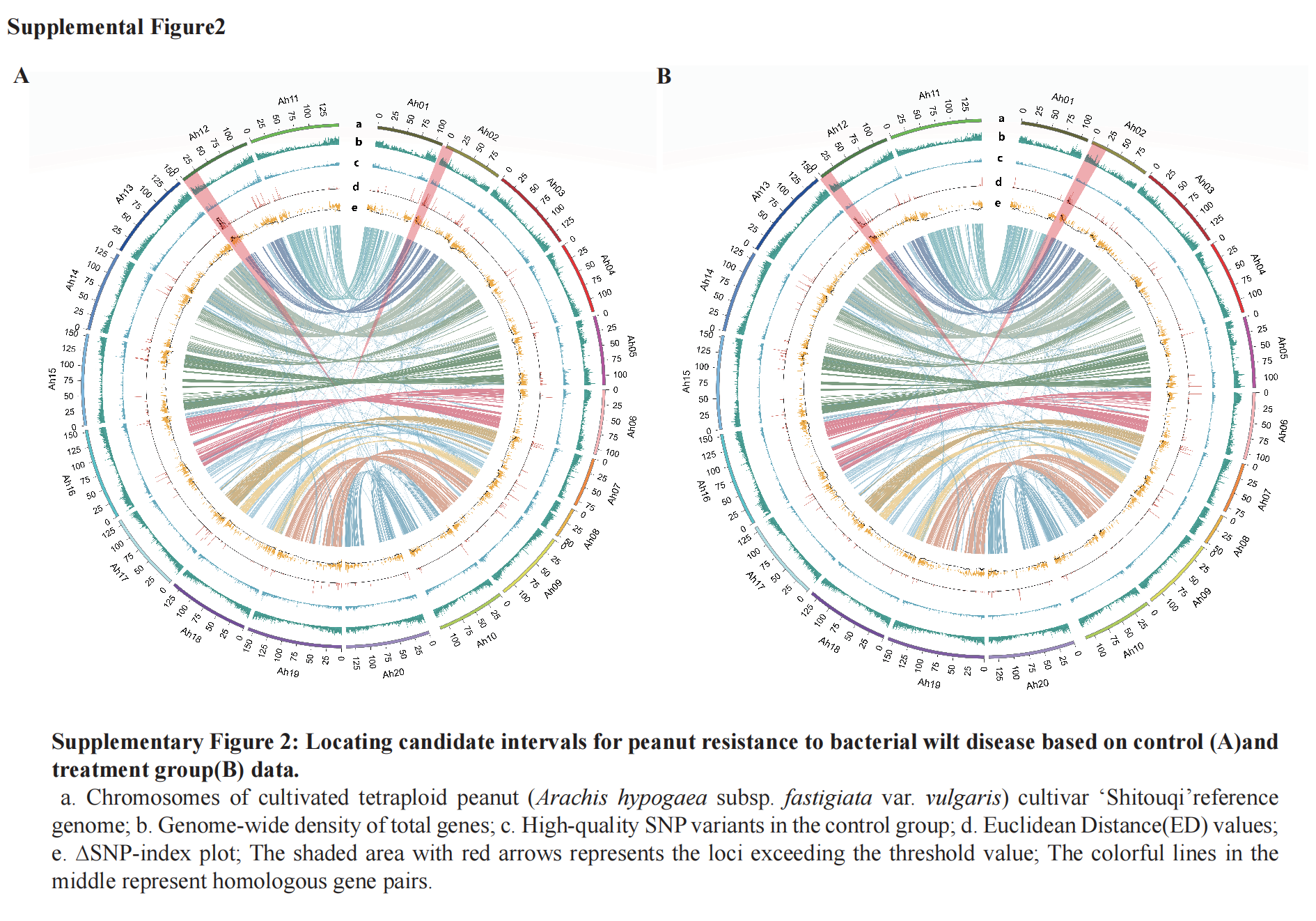

Supplement: Supplementary Figure 2 — Locating candidate intervals for peanut resistance to bacterial wilt disease based on control and treatment group data. a. Chromosomes of cultivated tetraploid peanut (Arachis hypogaea subsp. fastigiata var. vulgaris) cultivar ‘Shitouqi’reference genome; b. Genome-wide density of total genes; c. High-quality SNP variants in the control group; d. Euclidean Distance(ED) values; e. ΔSNP-index plot; The shaded area with red arrows represents the loci exceeding the threshold value; The colorful lines in the middle represent homologous gene pairs. [file Image2.tif]

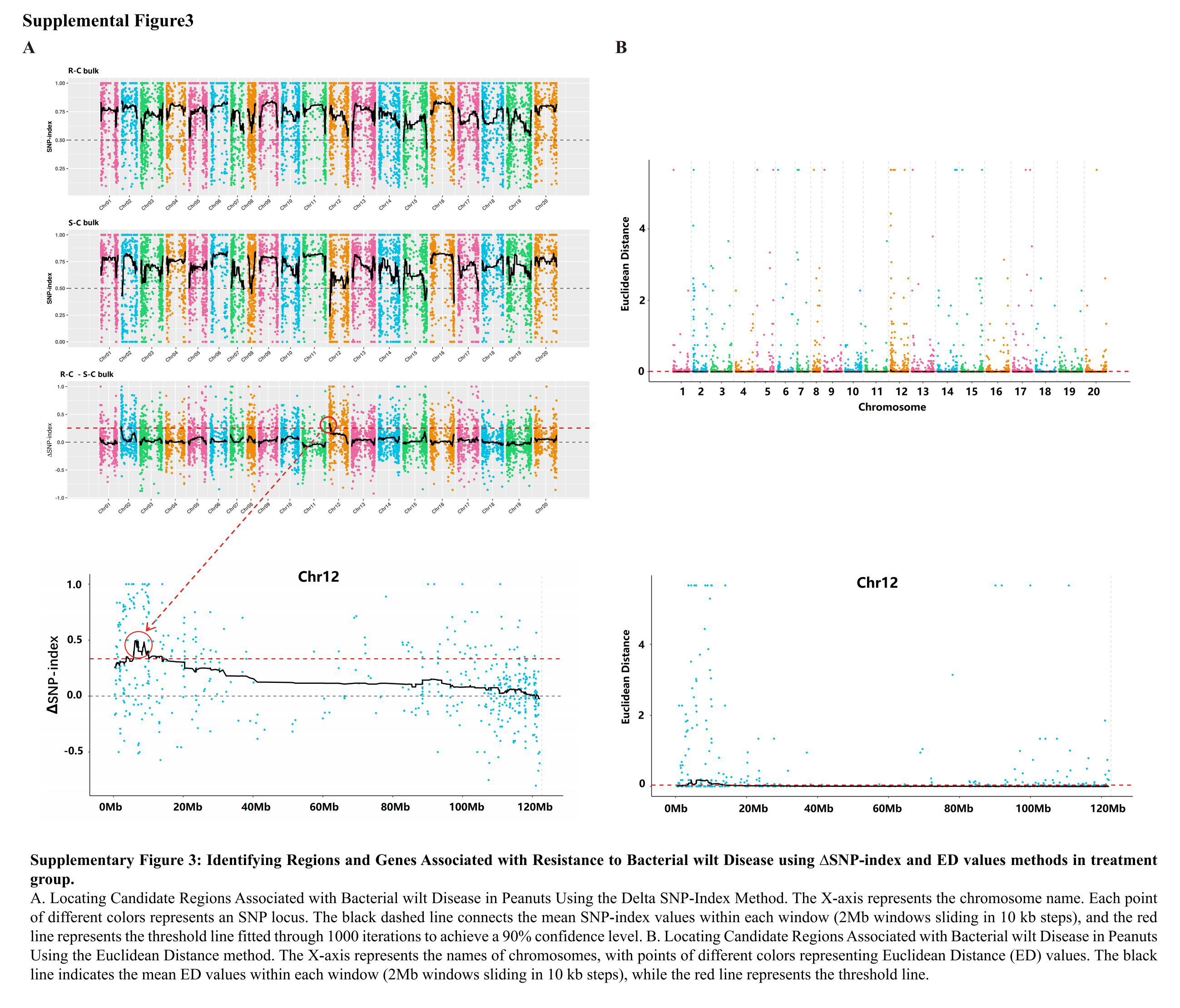

Supplement: Supplementary Figure 3 — Identifying Regions and Genes Associated with Resistance to Bacterial wilt Disease using ΔSNP-index and ED values methods in treatment group. (A). Locating Candidate Regions Associated with Bacterial wilt Disease in Peanuts Using the Delta SNP-Index Method. The X-axis represents the chromosome name. Each point of different colors represents an SNP locus. The black dashed line connects the mean SNP-index values within each window (2Mb windows sliding in 10 kb steps), and the red line represents the threshold line fitted through 1000 iterations to achieve a 90% confidence level. (B). Locating Candidate Regions Associated with Bacterial wilt Disease in Peanuts Using the Euclidean Distance method. The X-axis represents the names of chromosomes, with points of different colors representing Euclidean Distance (ED) values. The black line indicates the mean ED values within each window (2Mb windows sliding in 10 kb steps), while the red line represents the threshold line. [file Image3.tiff]

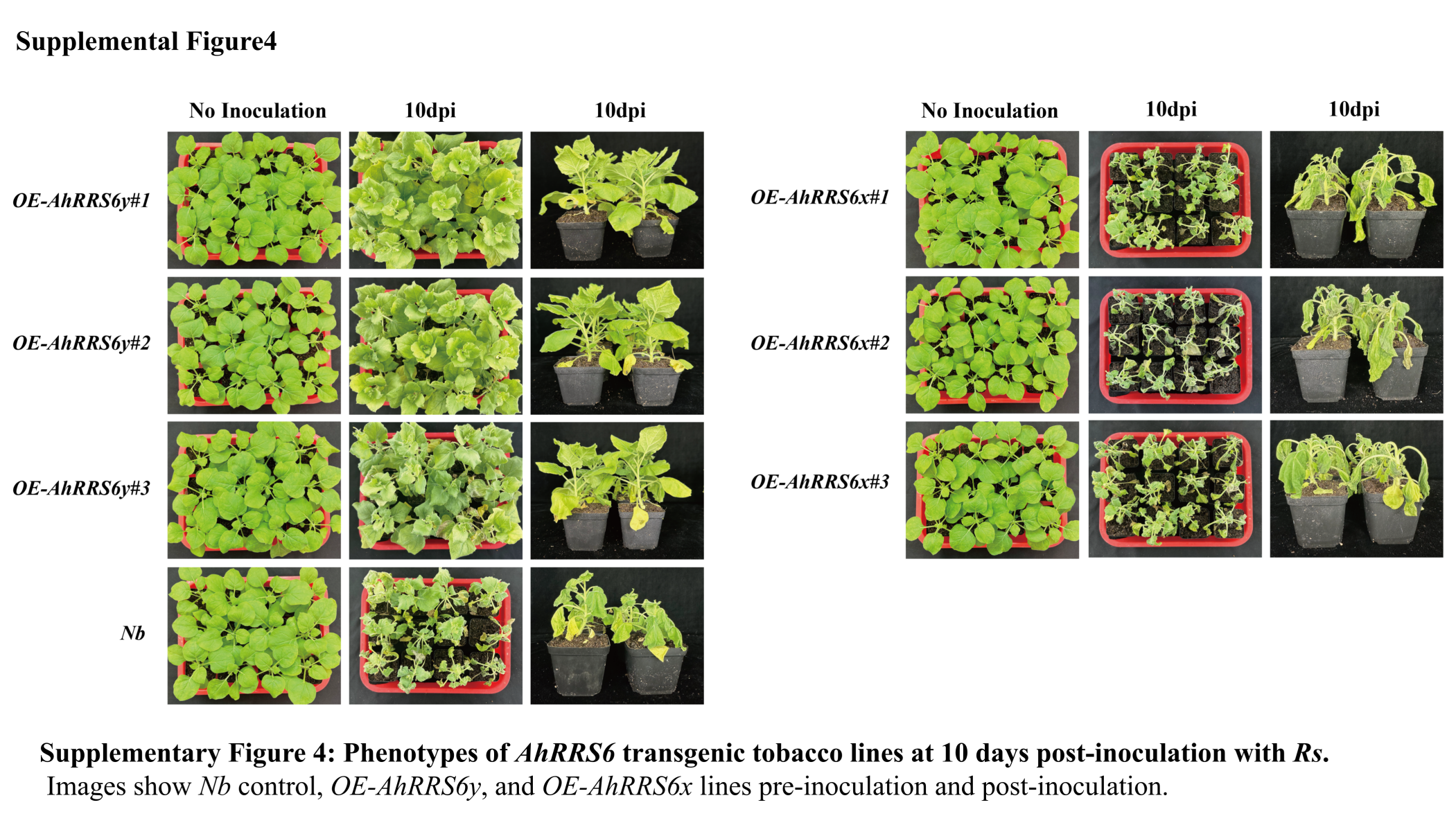

Supplement: Supplementary Figure 4 — Phenotypes of AhRRS6 transgenic tobacco lines at 10 days post-inoculation with Rs. Images show Nb control, OE-AhRRS6y, and OE-AhRRS6x lines pre-inoculation and post-inoculation. [file Image4.tiff]

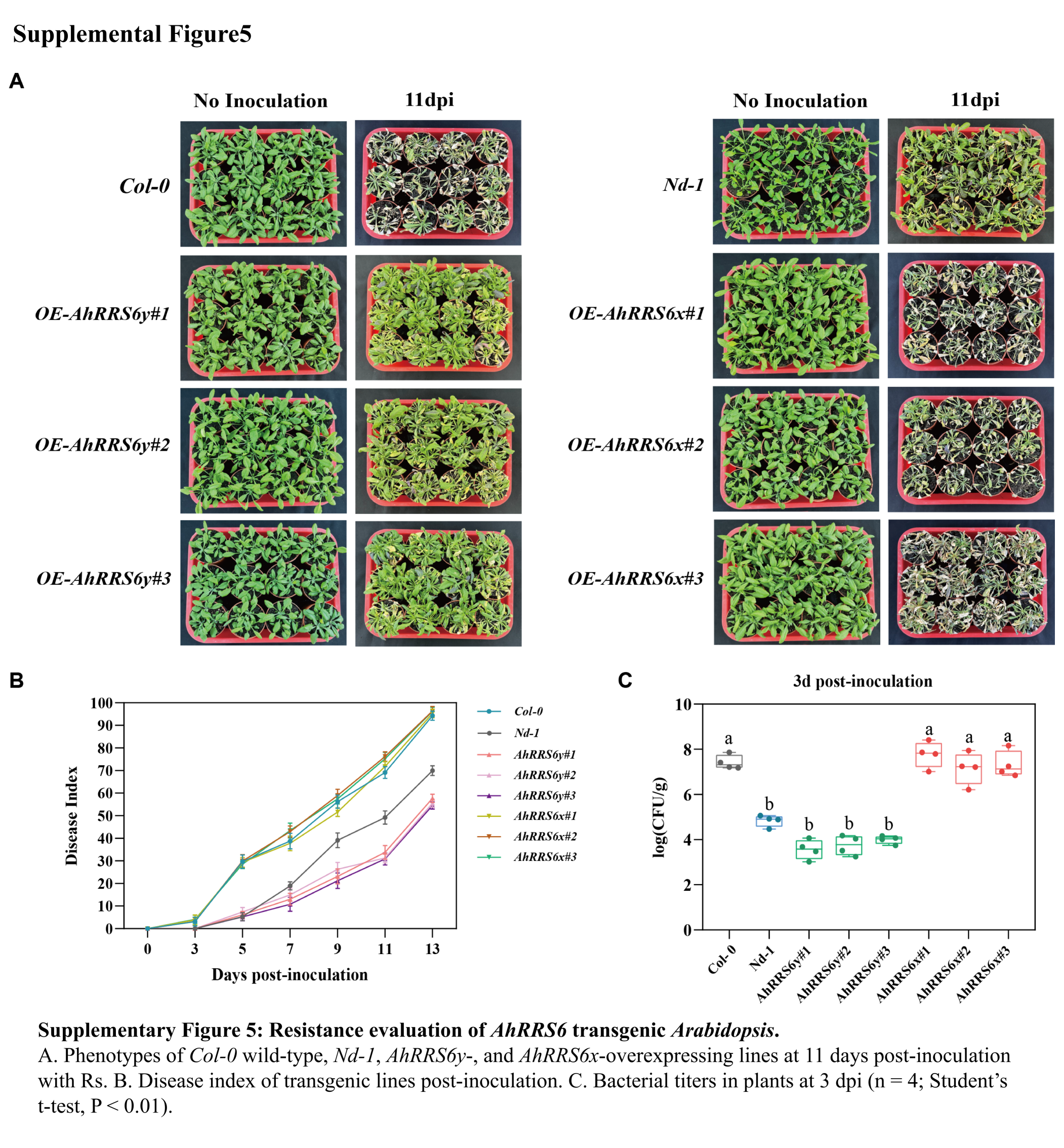

Supplement: Supplementary Figure 5 — Resistance evaluation of AhRRS6 transgenic Arabidopsis. (A). Phenotypes of Col-0 wild-type, Nd-1, AhRRS6y-, and AhRRS6x-overexpressing lines at 11 days post-inoculation with Rs. (B). Disease index of transgenic lines post-inoculation. (C). Bacterial titers in plants at 3 dpi (n = 4; Student’s t-test, P< 0.01). [file Image5.tiff]

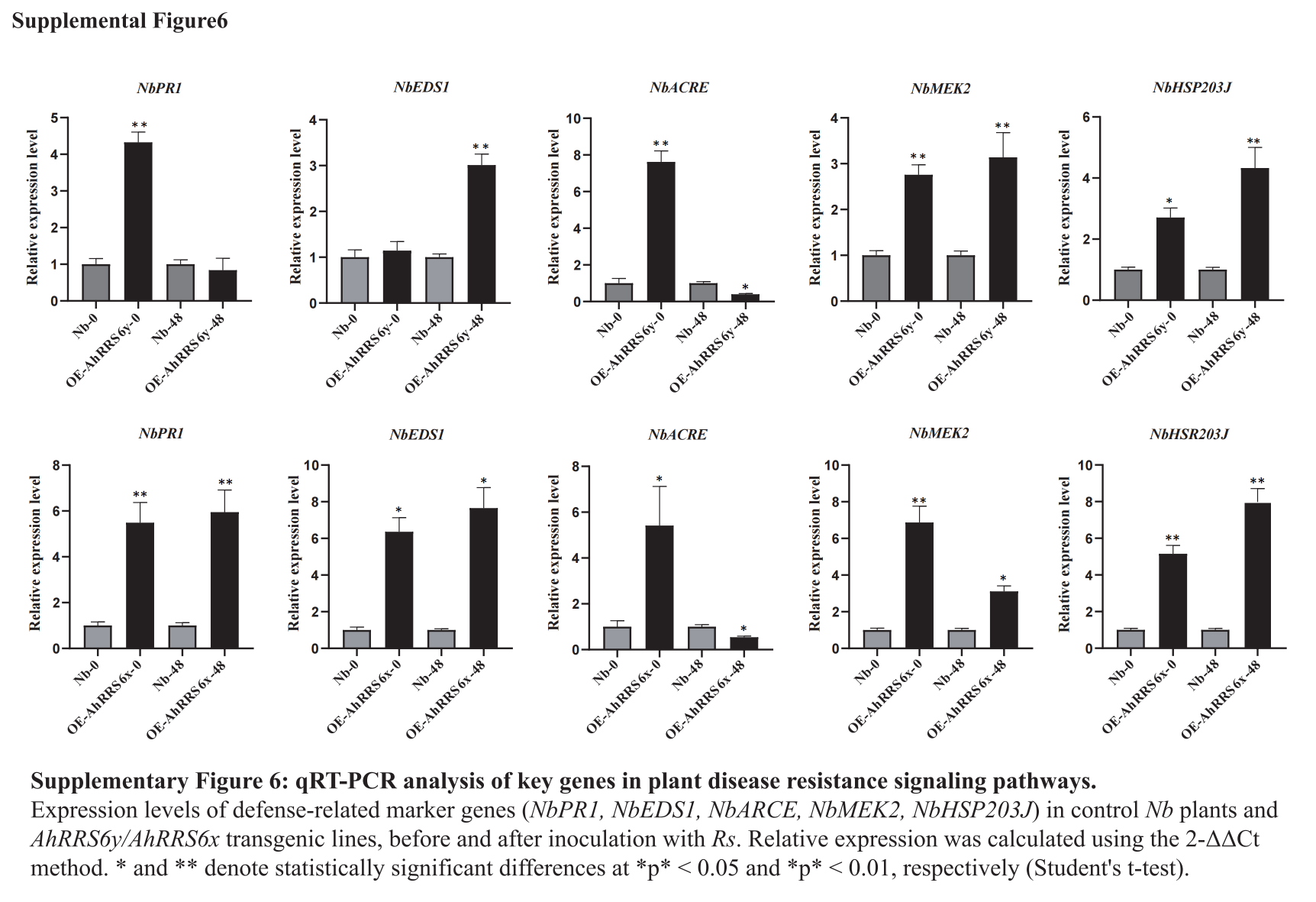

Supplement: Supplementary Figure 6 — qRT-PCR analysis of key genes in plant disease resistance signaling pathways. Expression levels of defense-related marker genes (NbPR1, NbEDS1, NbARCE, NbMEK2, NbHSP203J) in control Nb plants and AhRRS6y/AhRRS6x transgenic lines, before and after inoculation with Rs. Relative expression was calculated using the 2-ΔΔCt method. * and ** denote statistically significant differences at *p*< 0.05 and *p*< 0.01, respectively (Student’s t-test). [file Image6.tiff]

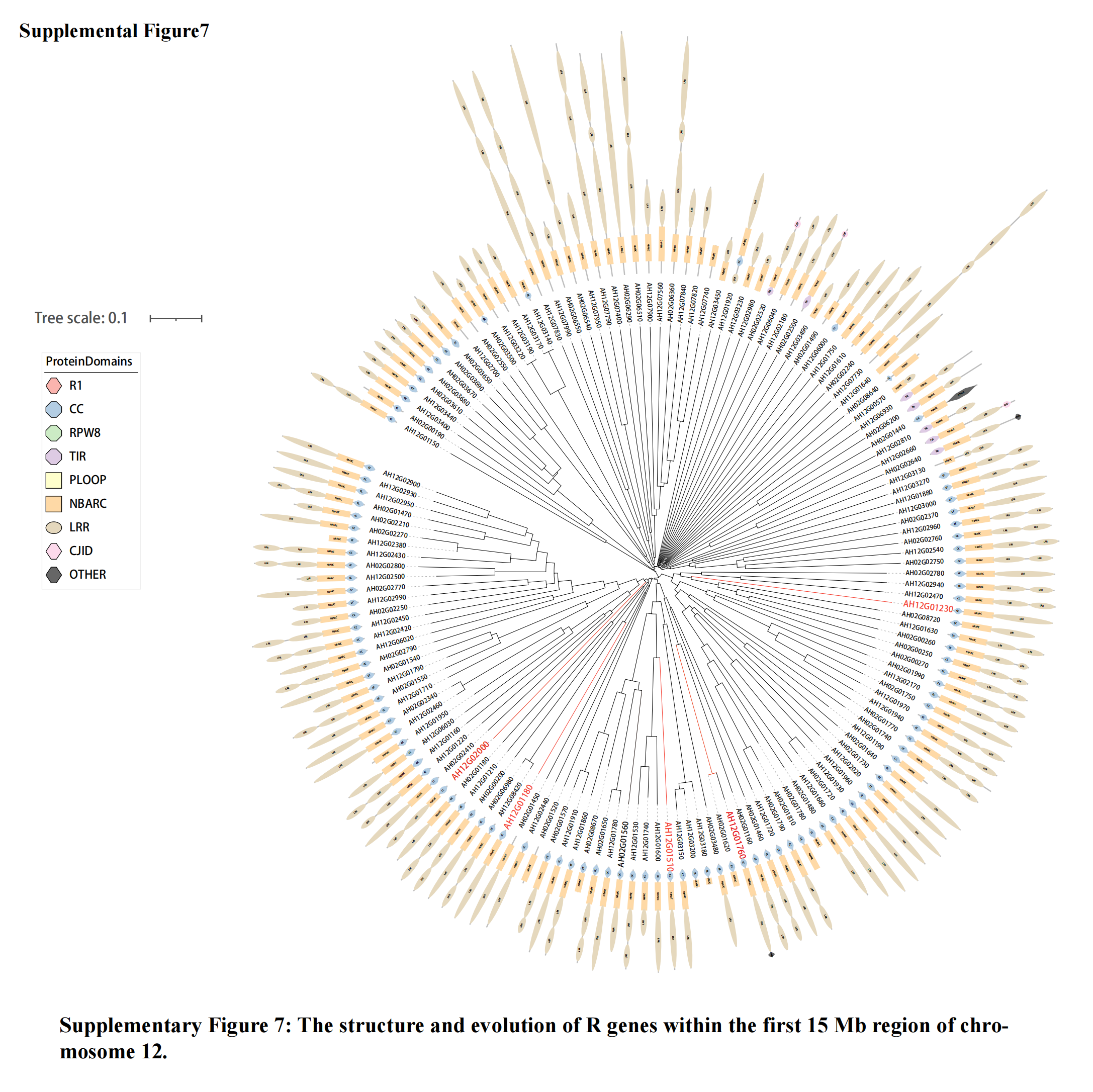

Supplement: Supplementary Figure 7 — The structure and evolution of R genes within the first 15 Mb region of chromosome 12. [file Image7.tif]
